# Product Selectivity Switching between CO and Formate in Electrocatalytic CO_2_ Reduction Driven by Ionic Liquid-Induced Reorientation of CO_2_ over Cathode

**DOI:** 10.1021/jacs.6c09949

**Published:** 2026-08-13

**Authors:** Yangshuo Li, Zhaojun Min, Mingming Sun, Huiyong Wang, Jianbo Yang, Qingwei Gao, Jianji Wang, Xiaoyan Ji

**Affiliations:** † Division of Energy Science, Luleå University of Technology, Luleå 97187, Sweden; ‡ Key Laboratory of Green Chemical Media and Reactions (Ministry of Education), Henan Key Laboratory for Green Media and Reactions, Henan International Joint Laboratory of Green Chemistry, Collaborative Innovation Center of Henan Province for Green Manufacturing of Fine Chemicals, School of Chemistry and Chemical Engineering, Henan Normal University, Xinxiang, Henan 453007, P. R. China; § College of Environmental and Chemical Engineering, Shanghai Key Laboratory of Materials Protection and Advanced Materials in Electric Power, 12596 Shanghai University of Electric Power, Shanghai 200090, P. R. China

## Abstract

Rationally switching reaction pathways to selectively produce diverse value-added chemicals is crucial for electrochemical CO_2_ reduction reactions (CO_2_RRs). However, such a goal is usually achieved by catalyst design, and it remains a challenge for electrolyte engineering. Herein, two structurally similar ionic liquids (ILs) differing only in the C2 substituent (H or –CH_3_) of the imidazolium cation, namely, 1-butyl-3-methylimidazolium hexafluorophosphate ([BMIM]­[PF_6_]) and 1-butyl-2,3-dimethylimidazolium hexafluorophosphate ([BMMIM]­[PF_6_]), were employed as electrolytes to modulate the product selectivity of CO_2_RR over a bimetallic Fe_5_Sn_1_NC electrocatalyst. It was found that CO_2_ reduction product was significantly switched between CO (Faradaic efficiency (FE) = 98.9%) and HCOOH (FE = 99.7%) via changing the IL cation from [BMMIM]^+^ to [BMIM]^+^ in the electrolyte, and the current density in the H-cell reached up to 93.7 mA·cm^–2^ at −2.2 V and 181.4 mA·cm^–2^ at −2.6 V (vs Ag/Ag^+^), respectively. Linear sweep voltammetry curves, H/D exchange, in situ/ex situ infrared spectroscopy, and density functional theory calculations suggest that the significant product switching between CO and HCOOH originates from the CO_2_ reorientation on the catalyst surface driven by different active sites in the imidazolium cations of the ILs, which leads to different reaction pathways in CO_2_RR.

## Graphical abstract

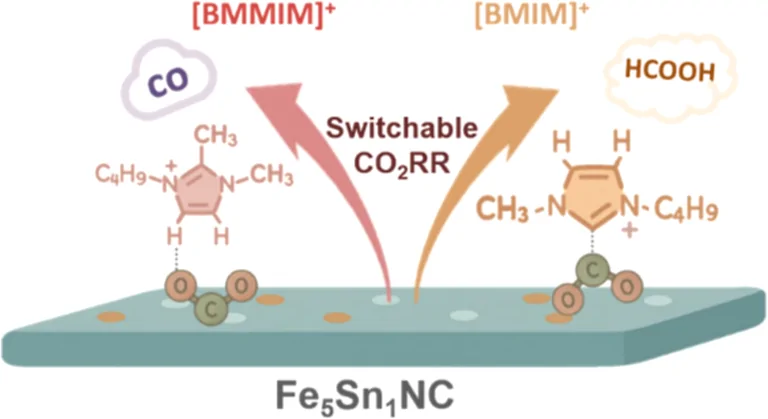

## Introduction

In recent years, electrochemical CO_2_ reduction reactions (CO_2_RRs) have been used to reduce carbon emissions and produce value-added chemical products simultaneously,^1^ which is of great importance for the sustainable development of modern civilized society. Among these products, CO and formic acid (HCOOH), as an important industrial intermediate and a high-energy-density liquid fuel, respectively, have been highlighted as the most commercially promising and industrially feasible products from CO_2_RR.^2^ However, CO_2_RR is an intricate process involving multiple proton–electron transfers and various active reaction intermediates at similar potentials, thus resulting in a number of side reactions and unsatisfactory product selectivity.^3,4^ In CO_2_RR, the adsorption and activation of CO_2_ are critical to the production of the target product. Principally, the carbon and oxygen atoms in CO_2_ represent the positive and negative charge centers of the CO_2_ molecule, respectively. Therefore, activation of CO_2_ at a metal (M) catalyst surface generally proceeds via two main modes: (i) π-backdonation of electrons from filled metal d-orbitals into the π antibonding orbital of CO_2_, predominantly localized on the C atom, which weakens the C=O bonds and increases the electrophilicity of the C center; (ii) σ-donation of lone electron pairs from the oxygen atoms of CO_2_ into empty orbitals of the metal, leading to M–O bond formation and concomitant polarization of the CO_2_ molecule.^5^ The coordination of the C atoms in CO_2_ tends to generate *COOH, which is the key intermediate for CO production.^6^ Instead, the coordination of the O atoms in CO_2_ leads to the formation of *OCHO as a fundamental intermediate to form HCOOH.^7,8^ Therefore, tailoring active sites of metal catalysts to modulate intermediate types and reaction pathways is critical for highly selective electrocatalytic production of target products from CO_2_.

In recent years, research has been carried out to manipulate the product distribution in CO_2_RR by tailoring the structure and morphology of catalysts and then regulating the formation of intermediates.^9–11^ For example, switching product selectivity from CO to HCOOH by adjusting the length of the Cu–S bond in the Cu_3_SbS_4_ catalyst^12^ or the morphology-induced local strain^13^ is well documented, and tuning the coordination structure of Cu in the Cu-based catalysts to switch the CO_2_RR product from C1 (such as CH_4_) to C^2+^ (such as ethylene and ethanol) is also reported.^14,15^ Nonetheless, it is generally difficult to precisely regulate the specific catalyst structure/morphology due to its complex synthesis procedures. Meanwhile, in such studies, aqueous carbonate solution is often used as the electrolyte, which suffers from low CO_2_ solubility, resulting in low CO_2_RR performance.^16^

In addition to catalyst design, developing electrolytes is also a promising strategy to boost product selectivity in CO_2_RR.^17,18^ It has been reported that under the catalysis of the same electrocatalyst, some electrolytes can not only enhance CO_2_ solubility but also reshape the reaction pathway to the target product, which makes it much easier to improve the selectivity and yield of the reaction products compared to the design of catalyst materials.^19^ In the advanced electrolytes reported previously, ionic liquids (ILs), especially imidazolium ILs, exhibited significant advantages in CO_2_RR due to their improved CO_2_ absorption and noticeable preactivation capability.^20,21^ For instance, Rosen et al.^22^ found that 1-ethyl-3-methylimidazolium tetrafluoroborate ([EMIM]­[BF_4_]) could reduce the overpotential for electrocatalytic CO_2_-to-CO conversion from 0.8 to 0.2 V due to the [EMIM]-CO_2_–BF_4_ adduct formation. Additionally, the system with IL was stable for 7 h of electrolysis to reach roughly 26000 turnovers. Han’s group^23^ reported that the selectivity of CH_4_ increased by 37.4% in a 1-butyl-3-methylimidazolium tetrafluoroborate ([BMIM]­[BF_6_])-based electrolyte compared with that in aqueous solution. It was also demonstrated that 1-ethyl-3-methylimidazolium bis­(trifluoromethylsulfonyl)­imid ([EMIM]­[Tf_2_N]) could remarkably inhibit the generation of oxalate but promote CO_2_RR to CO.^24^ Furthermore, Yuan et al.^25^ used 1-butyl-3-methylimidazolium hexafluorophosphate ([BMIM]­[PF_6_])-based electrolytes and metal Ag electrode with a relatively high active surface area of 495 cm^2^ to convert CO_2_ to CO in an upscaled CO_2_RR, the Faradaic efficiency (FE) was 83.9%, the reaction current was as high as 6.32 A, the competitive hydrogen evolution reaction was suppressed to only 2% FE, and the CO selectivity was 51.3% higher than in 0.1 M aqueous KHCO_3_. However, due to the unclear reaction mechanism and structure–property relationship of ILs in the CO_2_RR, switching the reduction product selectivity by engineering IL-based electrolytes is rarely reported so far.^26^

In this work, an attempt was made to switch the target product between CO and HCOOH from CO_2_ electrocatalytic reduction by only adjusting the structure of the IL in the electrolyte. As Fe and Sn are affinity sites for C and O,^27–30^ respectively, a bimetallic electrocatalyst of Fe_5_Sn_1_NC was developed. Meanwhile, the commonly used [BMIM]­[PF_6_] and 1-butyl-2,3-dimethylimidazolium hexafluorophosphate ([BMMIM]­[PF_6_]) ILs were chosen as the electrolytes, where the only difference in the structure of these ILs is that the C2–H in [BMIM]^+^ is replaced by a CH_3_ group in [BMMIM]^+^. The cationic structure of the ILs and the concept of product selectivity switching are shown in Scheme 1. It was discovered that these two ILs exhibited an extraordinary ability to switch the reduction product between CO and HCOOH by using the same catalyst of Fe_5_Sn_1_NC. In the 30 wt % [BMMIM]­[PF_6_]/acetonitrile (MeCN) electrolyte, the FE of CO (FE_CO_) was 98.9% with a current density of 93.7 mA·cm^–2^ at −2.2 V (vs Ag/Ag^+^), while in the 30 wt % [BMIM]­[PF_6_]/MeCN, the FE of HCOOH (FE_HCOOH_) was high up to 99.7% with a high current density of 181.4 mA·cm^–2^ at −2.6 V (vs Ag/Ag^+^) in a H-cell, which is the best performance reported so far based on the IL electrolytes in the H-cell (Table S1). Thus, the product selectivity between CO and HCOOH was readily switched by only replacing the C2–H of the IL cation with a methyl group. Furthermore, linear sweep voltammetry (LSV), H/D exchange, in situ attenuated total reflection surface-enhanced infrared absorption spectroscopy (ATR-SEIRAS), Fourier-transform infrared spectroscopy (FTIR), and density functional theory (DFT) calculations were combined to understand the microscopic reaction mechanism. It was revealed that such excellent reduction product switching between CO and HCOOH was ascribed to the imidazolium cation-induced reconstruction of CO_2_ adsorption configuration on the cathode surface. This approach provides a facile and flexible option to switch CO_2_RR product distribution by slightly tuning the cationic structure of the ILs.

**Scheme 1:**
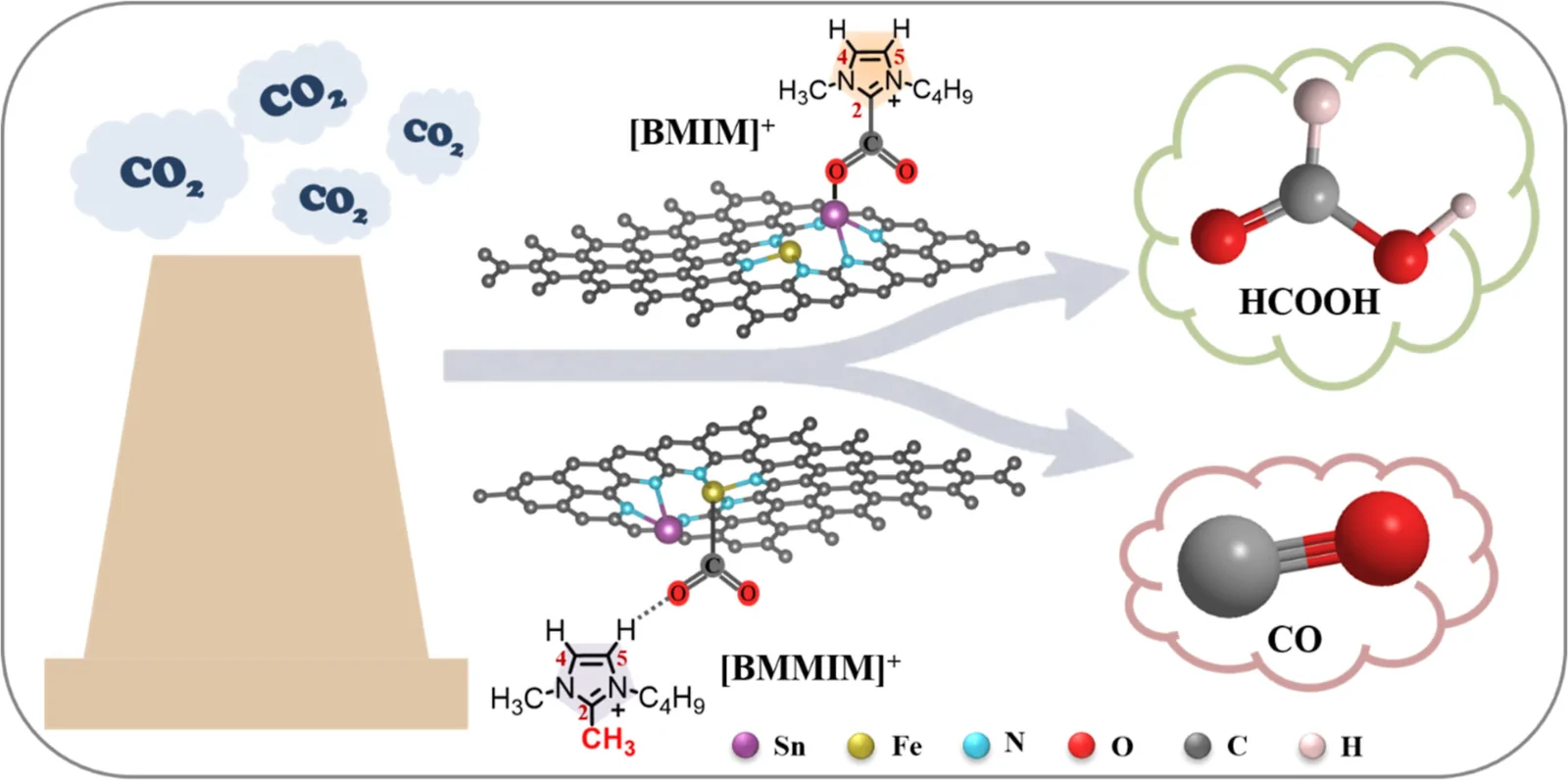
CO_2_RR Product Selectivity Switching in Different IL-Based Electrolytes

## Results and Discussion

Tin­(IV) chloride pentahydrate (SnCl_4_·5H_2_O), ferrous acetate (Fe­(C_2_H_3_O_2_)_2_), melamine, and carbon black were dispersed in ethanol via probe ultrasonication. The dispersion was heated at 800 °C for 2 h in a tube furnace under an Ar flow to prepare Fe_*x*_Sn_*y*_NC catalysts with varying Fe/Sn molar ratios (x/y). Details of the catalyst optimization process are provided in the Supporting Information. The actual Fe/Sn molar ratio was determined through inductively coupled plasma mass spectrometry (ICP-MS). The result is included in Table S2. Based on the results of ICP-MS, the optimized catalyst was designated as Fe_5_Sn_1_NC. Scanning electron microscopy (SEM) images showed that Fe_*x*_Sn_*y*_NC exhibited a similar morphology of small, fragmented structures with a size of 50–100 nm (Figure 1a and Figure S1). Their transparent stacked sheets could be clearly observed from the transmission electron microscopy (TEM) images (Figure S2). In the high-resolution TEM (HRTEM) image (Figure 1b), the flecks marked with orange circles are the loaded metals, and the only lattice fringes with an interplanar spacing of 0.39 nm was assigned to carbon black, suggesting the formation of amorphous Fe/Sn bimetallic with more active sites rather than the formation of nanoparticles with a crystalline structure.^31,32^ The selected area electron diffraction (SAED) pattern (Figure S3) further demonstrated the amorphousness of Fe_5_Sn_1_NC.

**Figure 1:**
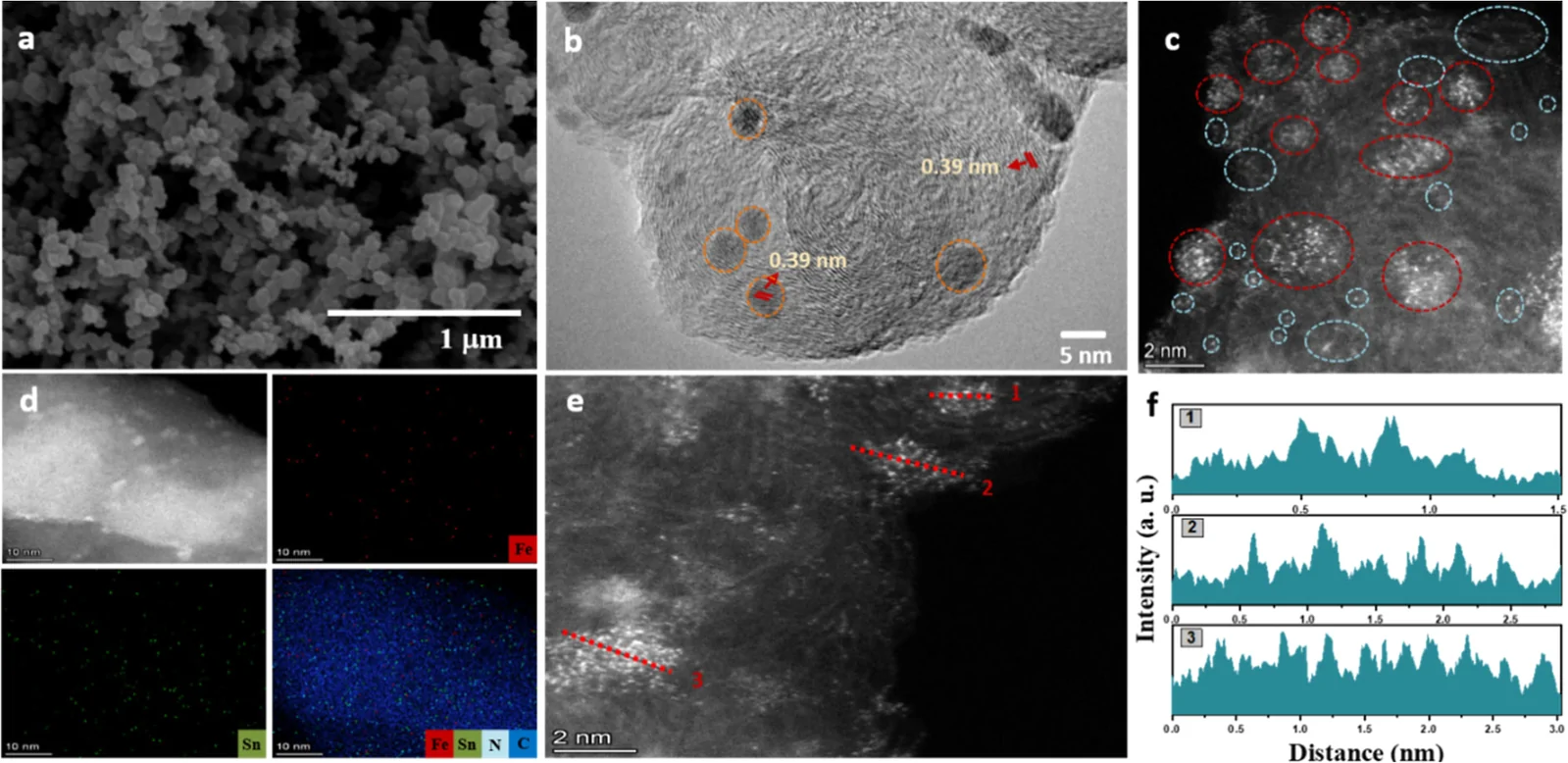
(a) SEM, (b) HRTEM, (c) high-angle annular dark-field scanning transmission electron microscopy (HAADF-STEM), and (d) STEM-EDX mapping images of Fe_5_Sn_1_NC. (e) Magnification of the HAADF-STEM image, where the single atomic Fe/Sn layer was highlighted by a red dashed line. (f) Line profiles extracted along the red dashed lines for the single atomic Fe/Sn layer in (e).

Furthermore, X-ray diffraction (XRD) patterns exhibited two most prominent peaks at 24.96° and 43.34° for each Fe_*x*_Sn_*y*_NC, corresponding to the (002) and (100)/(101) planes of carbon black,^31,33^ respectively (Figure S4). Separately, there are no evident metal diffraction peaks in the XRD pattern, indicating that Fe/Sn was loaded on the NC support without forming a crystal lattice. Further examination of Fe_5_Sn_1_NC by aberration-corrected HAADF-STEM revealed two types of Fe/Sn bimetal on the surface of the NC support: one was loosely arranged to form fully exposed atomic-layered clusters (circled in red) of 1–2 nm without obvious lattice fringes (Figure 1c), and the other was dispersed single atoms marked by blue circles in the adjacent area of the cluster. Energy-dispersive X-ray (EDX) spectroscopic mapping displayed uniformly distributed Fe (red) and Sn (green), as shown in Figure 1d. The intensity profile shown in Figure 1f corresponds to the HAADF intensity along the marked lines in the magnified HAADF-STEM image (Figure 1e). The relatively uniform intensity distribution and the absence of intensity features associated with multilayer stacking provide evidence for the single-atomic-layer configuration of the loaded Fe/Sn species. This structure maximizes the exposure of the Fe and Sn atoms, thereby generating highly accessible active sites.^34–36^ The electrochemical active surface area (ECSA) evaluated in the 30 wt % [BMMIM]­[PF_6_]/MeCN from electrochemical double-layer capacitance (C_dl_) derived from cyclic voltammetry (CV) measurements (Figure S5) noted that among the studied electrocatalysts, Fe_5_Sn_1_NC exhibited the highest C_dl_ value of 4.52 μF·cm^–2^, suggesting its most abundant active sites (Figure S6).

All CO_2_RR experiments were conducted in a standard three-electrode H-cell, with catalyst-coated carbon paper, Pt mesh, and Ag/Ag^+^ serving as the working, counter, and reference electrodes, respectively. Unless otherwise specified, all of the potentials reported in this work are the applied potentials without the *iR* correction, and the current density data were normalized to the geometric electrode area. The optimal Fe_5_Sn_1_NC catalyst was utilized to examine the influence of IL contents (0, 10, 30, and 50 wt %) in the electrolyte on the CO_2_RR performance. It was found that the FE_CO_ was the highest in the 30 wt % of [BMMIM]­[PF_6_] (Figure S7a). Although the total current density increased with the increase of [BMMIM]­[PF_6_] content (Figure S7b), 30 wt % [BMMIM]­[PF_6_]/MeCN was selected as the optimum electrolyte, taking into account both the FE_CO_ and the cost of the IL. Subsequently, CO_2_RR was conducted in 30 wt % [BMMIM]­[PF_6_]/MeCN and 30 wt % [BMIM]­[PF_6_]/MeCN, respectively, under the catalysis of Fe_5_Sn_1_NC. The contents of the produced HCOOH and the gaseous products were quantitatively determined through hydrogen nuclear magnetic resonance spectroscopy (^1^H NMR, Figure S8) and gas chromatography (GC, Figure S9), respectively. Surprisingly, it was found that in the 30 wt % [BMIM]­[PF_6_]/MeCN electrolyte, HCOOH was the main product with an excellent FE of 99.7% and a current density of 181.4 mA·cm^–2^ at −2.6 V (vs Ag/Ag^+^) (Figure 2a). Instead, when the C2–H in the imidazolium ring of the IL cation was replaced with –CH_3_, CO_2_ was almost entirely converted into CO, its FE was 98.9%, and the current density was 93.7 mA·cm^–2^ at −2.2 V (vs Ag/Ag^+^) (Figure 2b) in 30 wt % [BMMIM]­[PF_6_]/MeCN. Accordingly, the product switching between CO and HCOOH could be readily achieved by only replacing the C2–H in [BMIM]^+^ with a CH_3_ group. These results clearly indicate the pronounced effect of IL structure on the product selectivity in CO_2_RR and provide a simple but effective strategy for switching the distribution of reduction products by slightly changing the cationic structure of the ILs.

**Figure 2:**
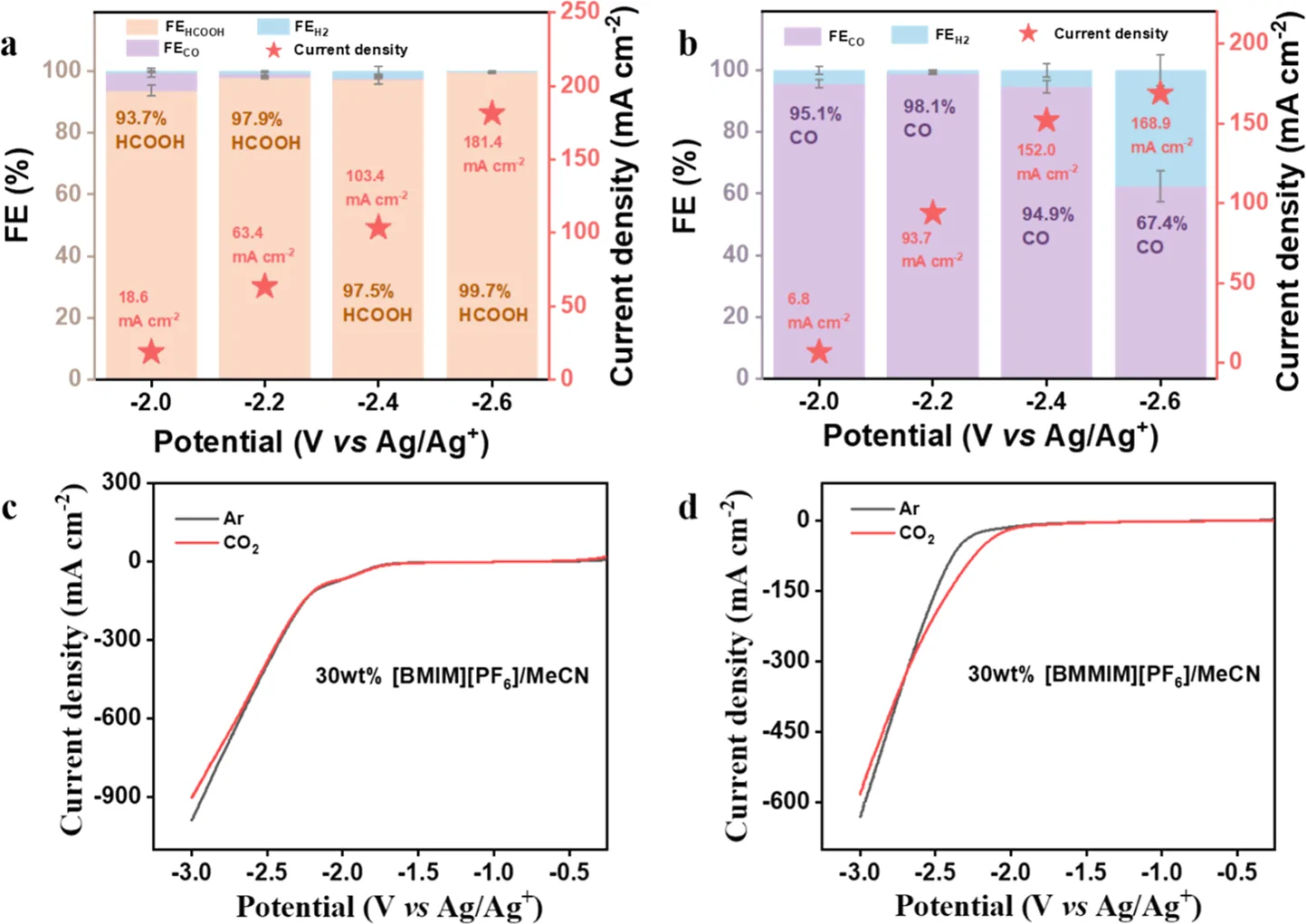
CO_2_RR performance of Fe_5_Sn_1_NC in (a) 30 wt % [BMIM]­[PF_6_]/MeCN and (b) 30 wt % [BMMIM]­[PF_6_]/MeCN at the potential from −2.0 to −2.6 V (vs Ag/Ag^+^), error bars represent the standard deviation of three independent measurements. LSV curves under Ar and N_2_, respectively, in (c) 30 wt % [BMIM]­[PF_6_]/MeCN, and (d) 30 wt % [BMMIM]­[PF_6_]/MeCN.

In order to confirm, the products were generated from the CO_2_RR rather than the decomposition of the electrolyte, the CO_2_RR was also performed by injecting ^13^C-labeled CO_2_ and Ar, respectively, in two IL-based electrolytes. It was shown that an obvious new peak at 161.6 ppm attributed to H^13^COO^–^ appeared in the ^13^C NMR spectrum (Figure S10a) of 30 wt % [BMIM]­[PF_6_]/MeCN after CO_2_ reduction. However, no reduction product was detected in the electrolyte through ^1^H NMR (Figure S10b) when the injected gas was changed to Ar. In the [BMMIM]^+^-based system, ^13^CO was also produced using labeled ^13^CO_2_ as the reactant, and its content was determined by GC-MS (Figure S11). Nevertheless, CO was not observed by GC (Figure S12) when injecting Ar rather than CO_2_ into the H-cell, indicating that all the products were from the CO_2_ reduction. Furthermore, the ^1^H NMR spectra (Figure S13) of the electrolytes remained essentially unchanged after the electrolysis, indicating that the structures of the imidazolium cations were chemically intact throughout the electrolysis. The water content before and after the electrolysis was also determined by the Karl Fischer titration. It was shown that water content increased from 0.13 to 3.93 wt % in the 30 wt % [BMMIM]­[PF_6_]/MeCN and from 0.26 to 4.38 wt % in the 30 wt % [BMIM]­[PF_6_]/MeCN after the electrolysis, indicating the occurrence of water crossover through the Nafion membrane during the electrolysis. Based on the report by Dongare et al.,^21^ the water content up to 1 vol % exerted slight influence on the onset potential and current response of CO_2_RR in the imidazolium-based IL electrolytes. Therefore, although the accumulated water may contribute to proton availability and hydrogen evolution at more negative potentials, the low water content is unlikely to significantly affect the initial electrochemical behavior. Additionally, a blank control experiment using the carbon black as the catalyst was conducted to confirm the negligible contribution of the carbon support to the observed catalytic activity. As shown in Figure S14, H_2_ was the dominant product in both IL-based electrolytes, indicating that the catalytic activity primarily originates from the metal active sites. Moreover, the concentrations of Fe and Sn in the 30 wt % [BMMIM]­[PF_6_]/MeCN solution were determined by ICP-MS. It was shown that the concentrations of Fe and Sn were below the detection limits (<0.02 mg/L and <3 μg/L, respectively) before and after the electrolysis, indicating negligible metal leaching during the electrolysis.

The different product distributions observed in the [BMIM]­[PF_6_] and [BMMIM]­[PF_6_] electrolytes suggest that the C2 site of the imidazolium cation plays an important role in determining the reaction pathway over the Fe_5_Sn_1_NC catalyst. However, the initial activation mechanism of CO_2_ in the imidazolium-based IL electrolytes remains in debate in the literature. While recent studies indicate that CO_2_ first receives an electron from the catalyst to form CO_2_^–^ and then interacts with the imidazolium cation,^37–39^ the previous reports suggest that the intermediate derived from the imidazolium cation may be formed prior to the activation of CO_2_.^40,41^ These different viewpoints may significantly depend on the nature of the catalysts. To explore the initial electrochemical behavior and understand the role of the imidazolium cations in regulating the product selectivity, LSV measurements were performed in 30 wt % [BMIM]­[PF_6_]/MeCN and 30 wt % [BMMIM]­[PF_6_]/MeCN under Ar and CO_2_, respectively. It was found that in the [BMIM]^+^-based electrolyte, the LSV curve under the CO_2_ atmosphere (Figure 2c) exhibits the same onset potential as that under the Ar-saturated conditions. By contrast, in the C2-blocked [BMMIM]^+^-based electrolyte, as shown in Figure 2d, the onset potential shifts toward the positive direction under CO_2_ compared with that under Ar. The distinct responses of these two electrolytes to CO_2_ indicate that the presence of CO_2_ can obviously affect the initial electrochemical activation process in the [BMMIM]^+^-based electrolyte, but only has a negligible effect in the [BMIM]^+^-based electrolyte. Since the C2 site is involved in the initial activation, this site may be the key for the markedly different product selectivity in these two electrolytes.

To further decode the role of these imidazolium ILs in switching the CO_2_RR products under the catalysis of Fe_5_Sn_1_NC (serves as cathode), a H/D exchange experiment was conducted in both electrolytes of 30 wt % [BMIM]­[PF_6_]/MeCN and 30 wt % [BMMIM]­[PF_6_]/MeCN under the Ar and CO_2_ atmosphere, respectively, by substituting the H_2_SO_4_/H_2_O anolyte with D_2_SO_4_/D_2_O. After 15 min of electrolysis, the electrolytes were collected with the same volume for the ^1^H NMR and ^2^H NMR spectroscopy measurements under identical conditions. It can be seen from Figure 3a–d that before electrolysis, no peaks of C2-D and C4/5-D in [BMIM]­[PF_6_] and C4/5-D in [BMMIM]­[PF_6_] were observed in the IL/MeCN electrolytes, which indicates that no obvious H/D exchange of reactive hydrogen occurred on the imidazolium cations under either Ar or CO_2_ atmosphere without electrolysis. However, after 15 min electrolysis at −2.0 to −2.8 V (vs Ag/Ag^+^), C2–H in [BMIM]­[PF_6_] was partially replaced by the deuterium atoms, but its C4/5-H/D exchange only occurred at lower potentials of −2.4 to −2.8 V (vs Ag/Ag^+^) under the Ar atmosphere (Figure 3a). This result suggests that C2–H is more reactive than C4/5-H in [BMIM]­[PF_6_] under electrolysis without CO_2_.

**Figure 3:**
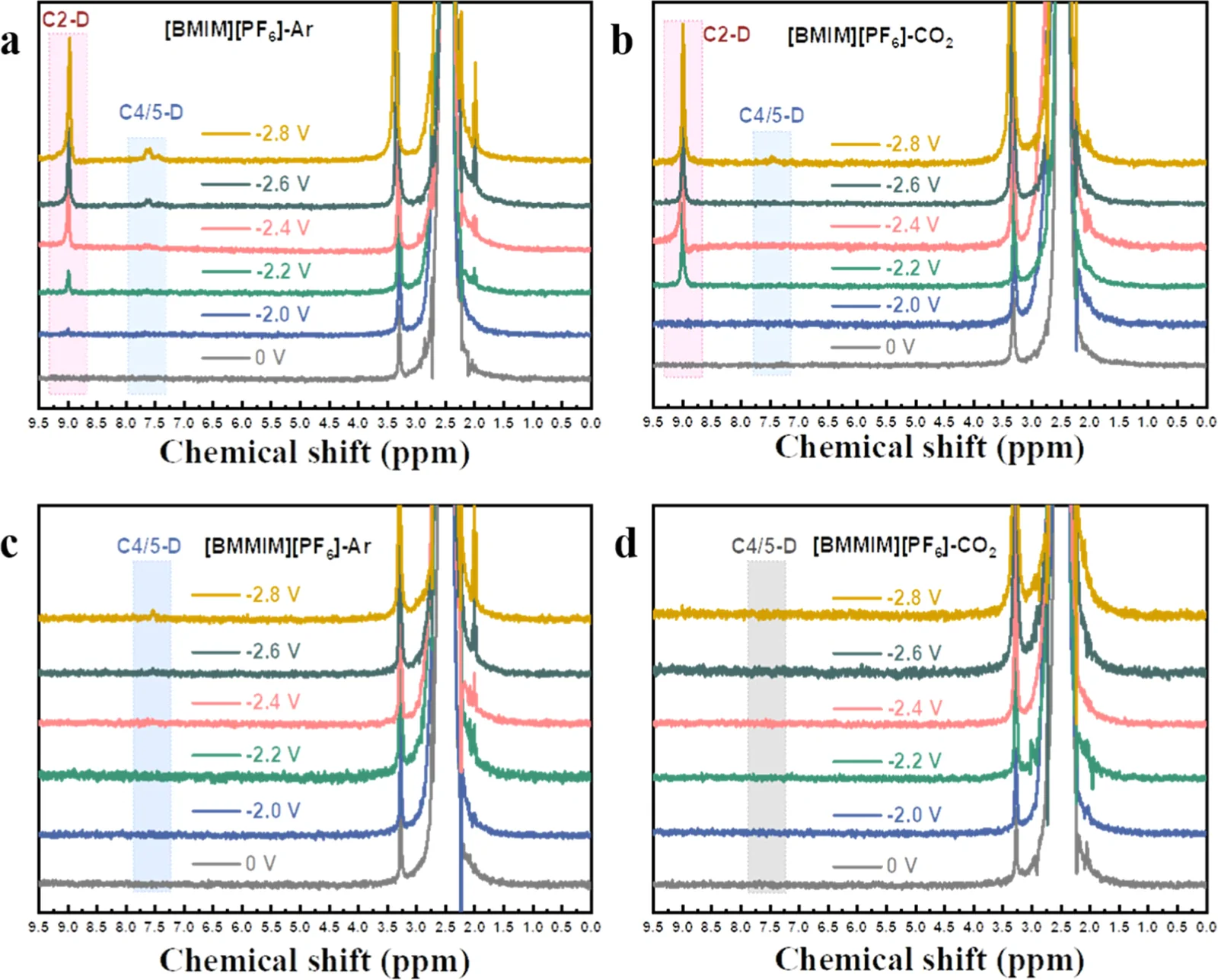
^2^H NMR spectra (in DMSO-*d*_6_) of the electrolytes: (a) 30 wt % [BMIM]­[PF_6_]/MeCN under Ar atmosphere; (b) 30 wt % [BMIM]­[PF_6_]/MeCN under CO_2_ atmosphere; (c) 30 wt % [BMMIM]­[PF_6_]/MeCN under Ar atmosphere, and (d) 30 wt % [BMMIM]­[PF_6_]/MeCN under CO_2_ atmosphere before (0 V vs Ag/Ag^+^) and after electrolysis (−2.0 to −2.8 V vs Ag/Ag^+^).

Upon the injection of CO_2_, the exchange of C4/5-H/D in [BMIM]­[PF_6_] was suppressed to a certain extent and was only observed at the relatively low potential of −2.8 V (vs Ag/Ag^+^). Instead, the peak of C2-D remained in a wider potential range under CO_2_ electrolysis (Figure 3b). This observation is consistent with the LSV results and further indicates that the presence of CO_2_ does not significantly alter the initial activation behavior of [BMIM]^+^. Therefore, we speculate that the C2–H of [BMIM]^+^ participates in the early stage activation process and tends to promote the formation of the highly active N-heterocyclic carbene (NHC), which subsequently interacts with the electron-deficient C atom of CO_2_.^38,41,42^ Simultaneously, when C4/5-H in [BMMIM]­[PF_6_] was also substituted by the deuterium atom at the lower potentials of −2.6 and −2.8 V (vs Ag/Ag^+^) under the Ar atmosphere (Figure 3c), this phenomenon disappeared in the CO_2_-saturated electrolyte (Figure 3d), signifying that the addition of CO_2_ inhibits the reduction of C4/5-H during the electrolysis, where C4/5-H is the only underlying active site to combine with the oxygen atom in CO_2_ through the hydrogen bond interaction.^42,43^ For the sake of comparison, all the ^1^H NMR spectra are presented in Figure S15.

According to previous reports,^44,45^ the pathways for the generation of HCOOH and CO bifurcate at the initial stage of CO_2_ adsorption on metal catalysts, determined by whether the C or O atom binds to the surface, as shown in Figure 4a. To investigate the role of [BMIM]­[PF_6_] and [BMMIM]­[PF_6_] in selectively switching between CO and HCOOH formation, in situ ATR-SEIRAS measurements were performed on the Fe_5_Sn_1_NC cathode to identify the reaction intermediates as the applied potential decreased from 0 to −3.0 V (vs Ag/Ag^+^). As illustrated in Figure 4b, the peaks at 1670 and 1633 cm^–1^ in the [BMIM]^+^-based system are assigned to the carbonyl (C=O) stretching of NHC–CO_2_ adduct and NHC–COOH species,^41,46,47^ respectively, which validates the adsorption configuration of the C atom in CO_2_ on the C2 site (NHC) of the IL cation during electrolysis. Notably, the characteristic peaks of key intermediate *OCHO at 1384, 1367, and 1335 cm^–1^ were observed in the [BMIM]^+^-based system, suggesting the CO_2_RR to HCOOH pathway and the adsorption of the O atom of CO_2_ on the Fe_5_Sn_1_NC in the [BMIM]^+^-based system.^48–50^ Moreover, the peak at 1605 cm^–1^ can be ascribed to the C=O stretching vibration of carboxylate,^51^ also indicating the reaction pathway to HCOOH. The weak vibrational peak in the range of 2087–2098 cm^–1^, assigned to CO* intermediate, appears at relatively high potentials and diminishes as the applied potential decreases (Figure 4b), suggesting that a small amount of CO was produced at high potential as a competitive product. The results align with those of the above-mentioned experimental CO_2_RR in the [BMIM]­[PF_6_]/MeCN electrolyte.

**Figure 4:**
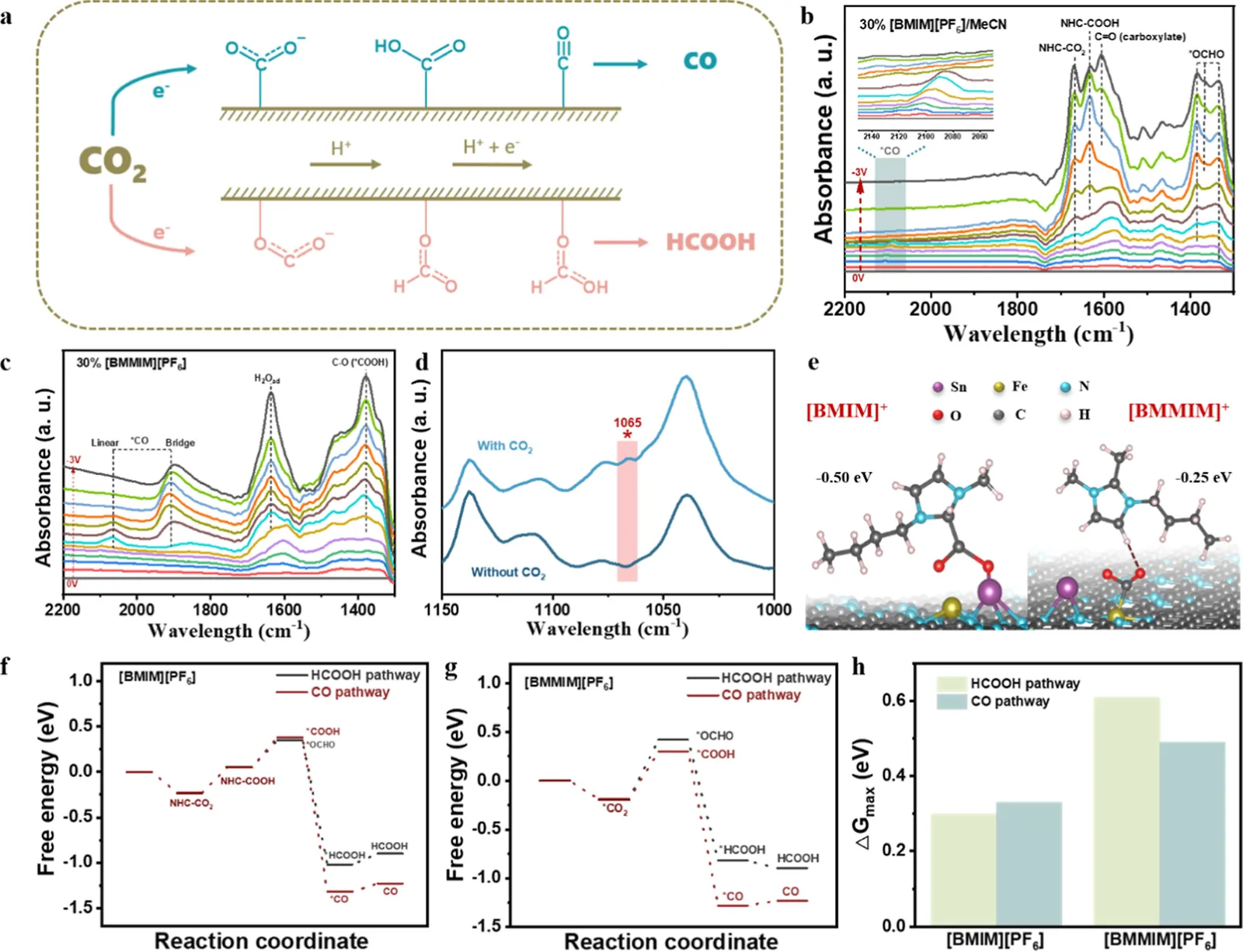
(a) The bifurcated pathway for the generation of COOH (red) and CO (blue) in the CO_2_RR and the corresponding CO_2_ adsorption configuration. In situ ATR-SEIRAS spectra of CO_2_RR in (b) 30 wt % [BMIM]­[PF_6_]/MeCN and (c) 30 wt % [BMMIM]­[PF_6_]/MeCN at the potential ranging from 0 to −3.0 V (vs Ag/Ag^+^). (d) FTIR spectra of 30 wt % [BMMIM]­[PF_6_]/MeCN with and without CO_2_. (e) The optimized CO_2_ adsorption configuration on the catalyst surface and the corresponding adsorption energy of CO_2_ on the catalyst in the presence of ILs. Gibbs free energy of CO_2_RR to CO and HCOOH from DFT calculations: (f) in [BMIM]­[PF_6_]/MeCN and (g) in [BMMIM]­[PF_6_]/MeCN. (h) Δ*G*_max_ for the CO_2_RR to CO and CO_2_RR to HCOOH pathways, respectively.

By contrast, in the in situ ATR-SEIRAS spectra of the [BMMIM]^+^-based system (Figure 4c), the peak at 1378 cm^–1^ is assigned to the key intermediate *COOH,^52,53^ while the peaks at 2064 and 1900 cm^–1^ are attributed to the C–O stretching vibration of the linear and bridged CO* intermediates,^54,55^ respectively. These intermediates imply the CO_2_RR-to-CO pathway in [BMMIM]­[PF_6_]/MeCN electrolyte, which is in agreement with the result observed in performance experiments and indicates the adsorption of carbon atom of CO_2_ on the Fe_5_Sn_1_NC. In addition, the peak at 1636 cm^–1^ stems from the adsorbed H_2_O molecules formed during the reduction reaction.^56^ Since the in situ ATR-SEIRAS measurements did not provide useful information to define the CO_2_ adsorption configuration influenced by the IL, the FTIR spectroscopy was used to probe the interaction between CO_2_ and [BMMIM]­[PF_6_]. It can be seen from Figure 4d that a new peak emerges at 1065 cm^–1^ after continuously injecting CO_2_ for 6 h in 30 wt % [BMMIM]­[PF_6_]/MeCN, which may be ascribed to the formation of a hydrogen bond between the O atom in the CO_2_ molecule and the C4/5-H in the imidazolium cation.^57,58^ Combined with the IR spectral evidence for the formation of an IL NHC–CO_2_ adduct, this result suggests that in the IL-based system, the orientation of the CO_2_ molecules on the Fe_5_Sn_1_NC cathode can be significantly influenced by the interaction of CO_2_ with C2 or C4/5 active sites on the imidazolium cation, thereby leading to different reaction pathways to CO and HCOOH in CO_2_RR.

DFT calculations were also implemented to demonstrate the crucial role of the imidazolium cation in driving the transformation of CO_2_ adsorption configuration on the catalyst surface. In the calculations, the structure of the Fe_5_Sn_1_NC catalyst was first optimized, and the result is exhibited in Figure S16, which is a two-dimensional plane similar to graphene, composed of hexagonal rings of carbon atoms. Within this framework, defect sites arise from the substitution of carbon by nitrogen in the carbon lattice, leading to the formation of strong covalent bonds with neighboring carbon atoms. Each nitrogen atom is coordinated to two adjacent carbon atoms, thereby forming part of a six-membered ring. Metal atoms (Fe and Sn) occupy the “vacancies” created by multiple nitrogen atoms to form coordination bonds with the surrounding three nitrogen atoms.^59,60^ The optimized catalyst model was then used in the following calculations.

As suggested by the preceding LSV and H/D exchange experiments, the presence of CO_2_ does not significantly alter the initial activation behavior of [BMIM]^+^. Together with the characteristic signals of the key intermediates identified by the in situ ATR-SEIRAS, it supports the hypothesis that [BMIM]^+^ may undergo electron transfer to form the NHC intermediate, and then subsequently interacts with CO_2_ and facilitates the HCOOH formation. To prove this hypothesis, the Gibbs free energies (Δ*G*) of “CO_2_ + e^–^” and “[BMIM]^+^ + e^–^” were calculated over the Fe_5_Sn_1_NC catalyst, respectively. The Δ*G*([BMIM]^+^+e^–^) of −2.51 eV is more negative than Δ*G*(CO_2_+e^–^) of −2.19 eV, suggesting that the electron transfer from the Fe_5_Sn_1_NC catalyst surface to [BMIM]^+^ is thermodynamically more favorable than the direct electron transfer to CO_2_. Therefore, [BMIM]^+^ is reasonably supposed to be electrolyzed into NHC by losing C2–H in the [BMIM]^+^-based electrolyte.

To determine how the CO_2_ molecule is adsorbed on the catalyst surface in the absence/presence of different ILs. All the possible structures are considered and exhibited in Figures S17–S19. Consequently, only the optimal CO_2_ adsorption configurations with/without IL and the corresponding adsorption energy were provided through DFT calculations. As shown in Figure S20, two CO_2_ adsorption configurations (A1 and A2) are observed in the absence of IL. In A1, CO_2_ is O-bound to Sn with an adsorption energy of −0.15 eV, while in A2, it is C-bound to Fe (−0.23 eV). Interestingly, as shown in Figure 4e, in the presence of ILs, only the A1 configuration is observed in [BMIM]^+^-based system (CO_2_ adsorption energy, −0.50 eV), and only the A2 configuration appears in the [BMMIM]^+^-based system (CO_2_ adsorption energy, −0.25 eV); the CO_2_ adsorption energies on the catalyst are both lower than those without IL, indicating more favorable and selective CO_2_ adsorption in the presence of ILs. This result suggests that depending on the structure of IL imidazolium cation, CO_2_ adsorption configuration on the catalyst is prone to be changed in the presence of ILs: the combination of the C atom in CO_2_ with NHC of the IL leads to the adsorption of the O atom in CO_2_ on the Sn atom in the [BMIM]^+^-based system, but the hydrogen-bond interaction between one of the O atoms in CO_2_ and the C5–H in [BMMIM]^+^ results in the C atom adsorption on the Fe atom in the [BMMIM]^+^-based system. Additionally, the distance between C5–H in the IL cation and the O atom in CO_2_ is 2.07 Å, as illustrated in Figure S21, suggesting the formation of a H-bond interaction.^61,62^ These results highlight the significant role of the imidazolium cations in tuning the CO_2_ adsorption configuration on the catalyst surface.

Nevertheless, previous studies showed that the spatial orientation of the imidazolium cations on the catalyst surface varies with applied potential. When the potential becomes more negative, the imidazolium cation tends to shift from a vertical orientation to a more parallel alignment relative to the catalyst surface.^16,21^ To evaluate the effect of the [BMIM]^+^ and [BMMIM]^+^ orientations on the CO_2_ adsorption configuration, the CO_2_ adsorption energy on the Fe_5_Sn_1_NC catalyst surface was also calculated in the presence of a parallel-oriented imidazolium cation relative to the catalyst surface. As shown in Figure S22, the CO_2_ adsorption energies in the presence of a parallel-oriented [BMIM]^+^ and [BMMIM]^+^ are −0.52 and −0.27 eV, respectively, which are still lower than those in the absence of the IL (−0.15 and −0.23 eV, respectively). This result suggests that the effect of the IL on the CO_2_ adsorption configuration is not strongly dependent on the spatial orientation of the imidazolium cation. On this basis, subsequent DFT calculations were carried out using static local adsorption configurations, where the potential-dependent dynamic reorientation of the imidazolium cations at the electrochemical interface was not considered.

To further elucidate the effect of cationic structure of the ILs on product selectivity, Δ*G* calculations were performed for the electrocatalytic CO_2_ reaction to produce HCOOH and CO in different IL-systems using various Fe/Sn-doped-monolayer-graphene-based explicit solvation models.^63,64^ For this purpose, the adsorption configurations of the reaction intermediates verified by in situ ATR-SEIRS and FTIR were first optimized on the Fe_5_Sn_1_NC catalyst surface. The intermediate structures for CO_2_RR-to-HCOOH in the [BMIM]^+^-based system and CO_2_RR-to-CO in the [BMMIM]^+^-based system are exhibited in Figures S23a and S24a, respectively. For comparison, the intermediate structures are also shown in Figures S23b and S24b. The corresponding Δ*G* values of CO_2_RR to CO/HCOOH over [BMIM]^+^ and [BMMIM]^+^ decorated Fe_5_Sn_1_NC catalyst are presented in Figures 4f-g, respectively. Clearly, the step from NHC–COOH to *COOH/*OCHO in the [BMIM]^+^-based system and that from *CO_2_ to *OCHO/*COOH in the [BMMIM]^+^-based system are the rate-determining steps, and the Δ*G* changes of the rate-determining step (Δ*G*_max_) are shown in Figure 4h. It is noted that the Δ*G*_max_ value of CO_2_RR-to-HCOOH (0.30 eV) is lower than that of CO_2_RR-to-CO (0.33 eV) over the [BMIM]^+^-decorated Fe_5_Sn_1_NC catalyst surface, while the Δ*G*_max_ of CO_2_RR-to-CO (0.49 eV) is lower than that of CO_2_RR-to-HCOOH (0.61 eV) over the [BMMIM]^+^-decorated Fe_5_Sn_1_NC catalyst surface. These results suggest that the pathway of forming HCOOH is more favorable in the [BMIM]^+^-based system, but the [BMMIM]^+^-decorated Fe_5_Sn_1_NC catalyst surface provides a more advantageous microenvironment for the CO production. These are in good agreement with experimental observations.

Finally, it should be emphasized that for a full understanding of the product switching from CO to HCOOH in the CO_2_RR, recognizing the role of the Fe and Sn atoms of Fe_5_Sn_1_NC in stabilizing the key intermediates is indispensable. To this end, additional DFT calculations were performed to determine the adsorption energies of key intermediates (*OCHO and *COOH) on the mono- and bimetallic catalysts, including FeNC, SnNC, and Fe_5_Sn_1_NC catalysts in the [BMIM]^+^ and [BMMIM]^+^ systems, respectively. The optimized structures of FeNC and SnNC are displayed in Figure S25. All of the possible adsorption configurations are considered as shown in Figure S26. It is found that *OCHO cannot be stably adsorbed on the monometallic catalysts in the [BMIM]^+^-based system. The adsorption energy of *OCHO on the Sn site in the presence of Fe (Figure S27a, −0.90 eV) is more negative than that on the Fe site in the presence of Sn (Figure S27b, −0.80 eV), indicating that *OCHO is preferentially adsorbed on the Sn site of Fe_5_Sn_1_NC. In the [BMMIM]^+^-based system, two *COOH adsorption configurations were optimized. In one configuration, *COOH is adsorbed on the Fe site in the absence of Sn, with an adsorption energy of −0.81 eV (Figure S28a). In the other configuration, *COOH is adsorbed on the Fe site adjacent to Sn, which exhibits a more negative adsorption energy of −1.05 eV (Figure S28b). This result suggests that the *COOH intermediate exhibits a stronger affinity for the Fe site, and the presence of Sn promotes *COOH adsorption on the Fe site. Collectively, bimetallic atoms are more efficient for the adsorption of two key intermediates in both systems. The charge density difference of Fe_5_Sn_1_NC with the adsorption of key intermediates (Figure S29) reveals significant charge transfer between Fe and Sn as well as between metal sites and the intermediates, indicating a synergistic effect of the bimetals of Fe_5_Sn_1_NC on the intermediates. Furthermore, the Bader charges of Fe and Sn in the catalyst before and after the adsorption of key intermediates were also calculated. The results are given in Table S6. It is noted that, upon *OCHO adsorption, the Sn site experiences a more pronounced electron depletion (+0.11), suggesting that Sn serves as the primary electron donor and is beneficial to the formate pathway. In contrast, during the *COOH adsorption, Fe undergoes a larger charge increase (+0.11), indicating its dominant role in stabilizing the carboxyl intermediates and favoring the CO pathway. These results demonstrate a site-specific bifunctional mechanism in Fe_5_Sn_1_NC, where Sn preferentially stabilizes the O-terminal of intermediates while Fe facilitates the stability of the C-terminal of intermediates.

Accordingly, the IL-manipulated mechanism of product switching over the Fe_5_Sn_1_NC catalyst was reasonably hypothesized, as illustrated in Figure 5. In the absence of IL, there are two possible adsorption configurations of CO_2_ on the Fe_5_Sn_1_NC surface: (i) oxygen-terminus adsorption of CO_2_ on the Sn atom and (ii) carbon-terminus adsorption of CO_2_ on the Fe atom. When using the ILs as electrolytes, imidazolium cations are first adsorbed on the cathode surface induced by the Coulomb force under electrolysis.^11^ The interaction of the imidazolium cations with CO_2_ reorients CO_2_ adsorption on the Fe_5_Sn_1_NC surface. In the [BMIM]­[PF_6_]/MeCN electrolyte, [BMIM]^+^ is supposed to undergo deprotonation upon gaining an electron to form a highly active NHC species, which is then readily to interact with the C atom of the CO_2_, resulting in the adsorption of the O atom in CO_2_ onto the Sn site of the Fe_5_Sn_1_NC cathode and the generation of the NHC–CO_2_ adduct. Afterward, the negatively charged oxygen atom attains an H^+^ to form NHC–COOH, and then the bond of NHC–COOH is broken after receiving an electron to generate the *OCHO intermediate. At the same time, NHC turns back into [BMIM]^+^ after capturing H^+^. After that, the *OCHO intermediate is subjected to proton–electron coupling, forming *HCOOH, followed by its desorption from the cathode surface, ultimately yielding HCOOH. On the other hand, although C2–H is absent in the [BMMIM]­[PF_6_]/MeCN electrolyte, its C4/5-H remains under electrolysis. C5–H combines with the oxygen atom in CO_2_ through a hydrogen bond interaction, leading to the adsorption of the C atom on the Fe site of the Fe_5_Sn_1_NC cathode surface. Under the effect of proton–electron coupling, *COOH is acquired and then undergoes proton–electron coupling again to produce the *CO intermediate and H_2_O. Ultimately, *CO is desorbed from the cathode surface to form the CO molecule. During these processes, the Sn site is more conducive to the stabilization of the *OCHO intermediate, while the Fe site favors the stabilization of the *COOH intermediate, and the cooperative effect of the bimetallic sites enhances the CO_2_RR performance. Therefore, by slight changes in the structure of the imidazolium cation of the ILs, product switching between CO and HCOOH can readily be achieved.

**Figure 5:**
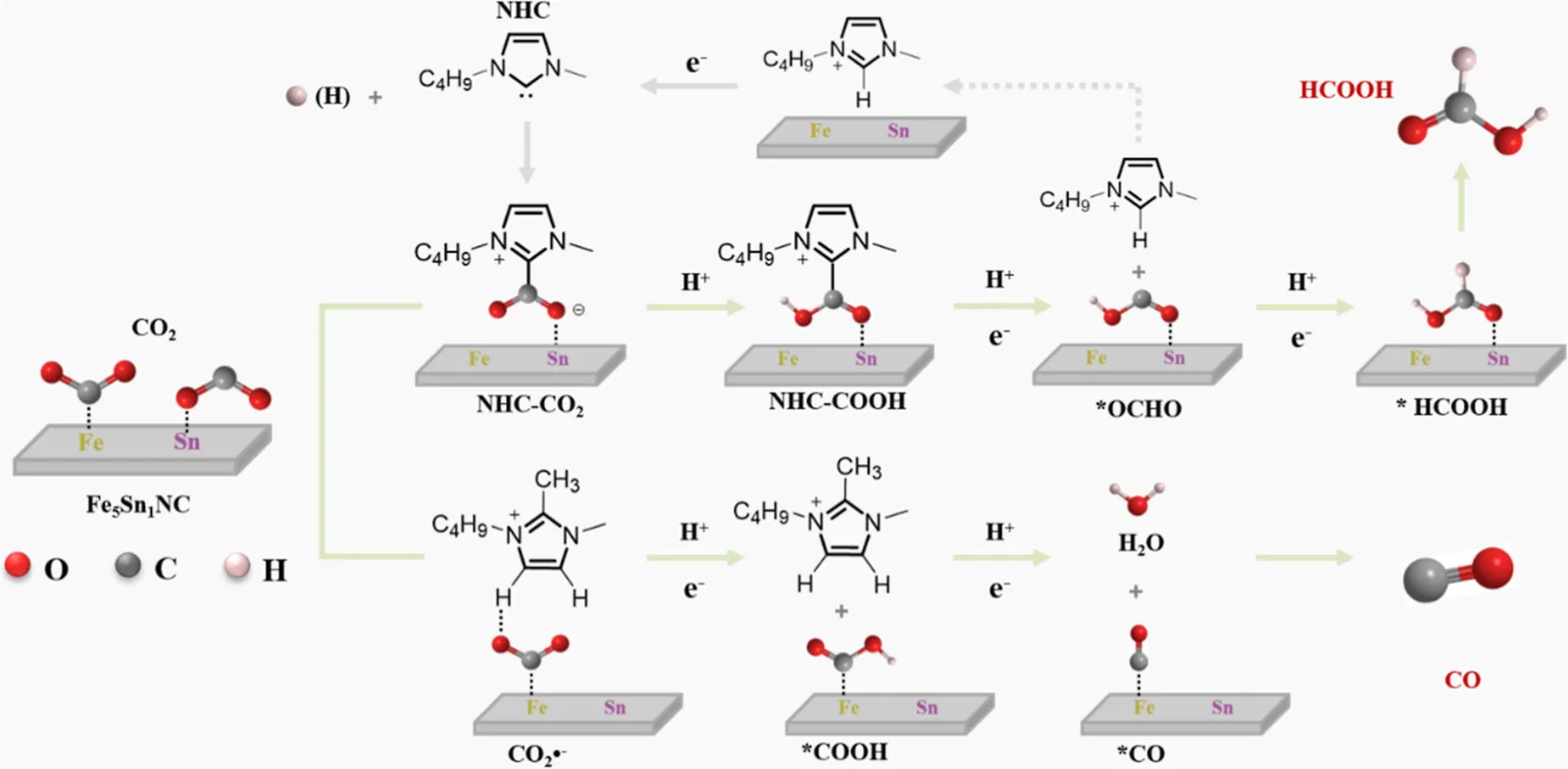
Hypothesized product switching mechanism in CO_2_RR over Fe_5_Sn_1_NC catalyst tuned by [BMIM]­[PF_6_] and [BMMIM]­[PF_6_].

## Conclusion

In summary, imidazolium ILs with different active sites were designed as electrolytes to switch the product selectivity in the CO_2_RR. It was found that a small difference in the IL cation structure would result in significantly different electroreduction reaction pathways to achieve different products over the same cathode of Fe_5_Sn_1_NC. During electrolysis, C2–H in the imidazolium cation of [BMIM]­[PF_6_] tends to be reduced, leading to the generation of a highly active NHC carbene with a lone pair of electrons, which was inclined to bind with the positive charge center of the carbon atom in CO_2_, thus activating CO_2_ and directing CO_2_RR to the HCOOH pathway. On the other hand, when C2–H in [BMIM]^+^ was replaced by –CH_3_ to produce [BMMIM]^+^, the reduction of C4/5-H in [BMMIM]­[PF_6_] was suppressed by CO_2_ during the electrochemical reduction reaction. As a result, C5–H was stable and tended to combine with the oxygen atom of CO_2_ through hydrogen-bonding interactions. Under these circumstances, the adsorption configuration of the CO_2_ molecule on the catalyst surface would be substantially affected by the interactions between the IL and CO_2_, thereby leading to different key intermediates. Consequently, the product could be significantly switched from HCOOH (FE_COOH_ = 99.7%) to CO (FE_CO_ = 98.9%) via changing the IL from [BMIM]­[PF_6_] with the main active site of C2–H to [BMMIM]­[PF_6_] with the primary active site of C4/5-H. Furthermore, the mechanism studies from the LSV, H/D exchange, the in situ ATR-SEIRAS and FTIR measurements, and theoretical calculations indicate that the Sn site preferentially stabilizes the *OCHO intermediate, whereas the Fe site favors the stabilization of the *COOH intermediate, and the synergistic effect of the bimetallic sites enhances the stabilization of key intermediates. Therefore, the oxygen-end adsorbed CO_2_ on the Sn site caused via [BMIM]^+^ led to the pathway toward the HCOOH production in the [BMIM]^+^-based system, while the carbon-end adsorbed CO_2_ on the Fe site influenced via [BMMIM]­[PF_6_] electrolyte resulted in the formation of CO with [BMMIM]­[PF_6_] electrolyte. Overall, this finding opens a new pathway for product switching in the CO_2_RR and provides deeper insights into the fundamental function of ILs in the CO_2_RR.

## Supplementary Material



## Notes

Experimental details, DFT calculation details, SEM images, TEM images, SAED pattern, XRD pattern, CV curves, NMR spectra, GC-MS spectra, theoretical simulation results, product distributions, and additional tables (PDF)
