# Supplementary material for: Product Selectivity Switching between CO and Formate in Electrocatalytic CO2 Reduction Driven by Ionic Liquid-Induced Reorientation of CO2 over Cathode
Source: J Am Chem Soc. 2026 Aug 13;148(33):35983–94. doi: 10.1021/jacs.6c09949 (PMC13523762; doi:10.1021/jacs.6c09949)
Supplement: Supplementary file 1 [file ja6c09949_si_001.pdf]

Supporting information

Product Selectivity Switching Between CO and Formate in Electrocatalytic CO_2 Reduction Driven by Ionic Liquid Induced Reorientation of CO_2 Over Cathode

Yangshuo Li,[a] Zhaojun Min,[b] Mingming Sun,[b] Huiyong Wang,[b] Jianbo Yang,[c] Qingwei Gao,*[c] Jianji Wang*[b], Xiaoyan Ji*[a]

[a] Division of Energy science
Luleå University of Technology
Luleå 97187, Sweden
E-mail: xiaoyan.ji@ltu.se

[b] Key Laboratory of Green Chemical Media and Reactions, Ministry of Education
Henan International Joint Laboratory of Green Chemistry
Collaborative Innovation Center of Henan Province for Green Manufacturing of Fine Chemicals
School of Chemistry and Chemical Engineering
Henan Normal University
Xinxiang, Henan 453007, China
E-mail: jwang@htu.edu.cn

[c] College of Environmental and Chemical Engineering
Shanghai Key Laboratory of Materials Protection and Advanced Materials in Electric Power
Shanghai University of Electric Power
Shanghai 200090, China
E-mail: qingwei.gao@shiep.edu.cn

Table of contents

1. Experimental Section S1

2. Figures S1-S29 ....S5

3. Tables S1-S6 ....S17

4. References S19

1. Experimental Section

Chemicals

Ferrous acetate ($Fe(C_2H_3O_2)_2$, 95%) was purchased from Bidepharm (China), tin(IV) chloride pentahydrate ($SnCl_4•5H_2O$, 99%) and melamine ($C_3H_6N_6$, 99%) were bought from Alladin (China). Carbon black, 1-butyl-3-methylimidazolium hexafluorophosphate ([BMIM][PF_6], 97%) and 1-butyl-2,3-dimethylimidazolium hexafluorophosphate ([BMMIM][PF_6], 97%) were attained from Macklin (China). Sulfuric acid (H_2SO_4, 95.0-98.0%) and acetonitrile (MeCN, ⩾99.0 %) were obtained from Chengdu Chron Chemical Co., Ltd. (China). $^{13}CO_2$ gas (enrichment, 99 at.%, Sigma-Aldrich Co., Ltd) and Nafion solution (5.0 min 5.4 max wt%) were bought from Suzhou Sinero Technology Co.,Ltd (China), and Nafion 117 membrane was required from Dupont Co. Ltd (China). All the chemicals were used without further purification unless otherwise specified.

Synthesis of Fe_xSn_yNC catalysts

Fe_xSn_yNC was synthesized by first mixing a certain amount of $Fe(C_2H_3O_2)_2$, $SnCl_4$ $•5H_2O$, and 0.2 g $C_3H_6N_6$ and 0.1 g carbon black in the ethanol, and then subjected to sonication for 2 hours. After that, the suspensions were centrifuged at 18000 rpm for 5min to remove the supernatant. The precipitates were treated with thermal annealing at the temperature changed from 700°C to 800°C, 900°C and 1000°C for 2 hours under the flow Ar atmosphere to produce a series of distinct Fe_xSn_yNC as the CO_2RR catalysts. The temperature increased from 20°C to 700, 800, 900 and 1000 °C, respectively, at the rate of 2 °C/min. FeNC and SnNC were prepared similarly by adding only the corresponding metal precursor.

Before estimating the performance of CO_2RR, the catalyst preparation parameters were optimized systematically. (1), the feed molar ratio of the “metal-to-support” was

examined at an arbitrary selected Fe/Sn molar ratio of 1:1. The optimal feed molar ratio of "metal (Fe and Sn) to support" was determined as (1:1):100 (Table S3). (2), based on (1), the feed molar ratio of Fe-to-Sn (i.e., Fe/Sn) was subsequently changed to find out an optimal value. It was interesting to note that the optimal feed molar ratio of Fe-to-Sn was still 1:1 (Table S4). (3), the synthesis temperature was optimized using the identified optimal parameters, and the best catalytic performance was achieved at 800 °C (Table S5).

Characterizations of the catalysts

The mass percentage of metal elements was determined by inductively coupled plasma-mass (ICP-MS) on an Agilent 7800 (MS) spectroscopy. Scanning electron microscopy (SEM) images were obtained from a SU8010 field-emission scanning electron microscope (Hitachi Company) operated at 5 kV acceleration voltage. The transmission electron microscopy (TEM), high-resolution TEM (HRTEM) images and the selected area electron diffraction (SAED) patterns were observed on a JEM-2100F transmission electron microscope with two silicon drift detectors at 200 kV (Japan Electron Optics Laboratory, Japan). The X-ray diffraction (XRD) patterns were measured by a D8 & Advance X-ray diffractometer (Bruker Company, 40 kV, 40 mA) with Cu Kα radiation (λ = 1.5418 Å) as X-ray excitation source. The aberration-corrected high-angle annular dark-field scanning transmission election microscopy (HAADF-STEM) images and energy-dispersive X-ray spectroscopy (EDX) mapping were recorded on an American FEI Spectra 300 (Super C) at 300 KV. The liquid products were detected by ^{1}H NMR spectroscopy (Bruker AVIII HD 600 instrument at 600 MHz), and the gaseous products were analyzed through a GC9790II gas chromatography (GC, FULI, China) equipped with a thermal conductivity detector (TCD) and a flame ionization detector (FID) simultaneously. The gaseous products from $^{13}CO_2$ source were identified via gas chromatography-mass spectrometry (GC-MS, Agilent GC/MS 7000D). The in-situ attenuated total reflection surface-enhanced infrared absorption spectroscopy (ATR-SEIRAS) spectra were recorded on a Bruker

VERTEX 70v vacuum in situ Fourier infrared spectrum analyzer. Fourier-transform infrared spectroscopy (FTIR) spectra were scanned on a Perking Elmer Spectrum 400F spectroscopy from 4000 to 400 cm^{-1} at room temperature.

Electrochemical measurements

All the CO_2RR measurements were performed in the H-cell with a standard three-electrode system using an electrochemical workstation (CHI 660E, Shanghai CH Instruments Co., China). Working electrode was the catalyst ink coated on carbon paper with the area of 0.5*0.5 cm^2. The catalyst ink was prepared through mixing 1 mg catalyst powder, 350 μL deionized water, 600 μL isopropanol and 50 μL Nafion together and then ultrasonicating for 30 min. Ag/Ag^+ (0.01 M $AgNO_3$ and 0.1 M tetrabutylammonium perchlorate in MeCN solution)[(1)] and Pt mesh was used for the reference and counter electrodes, respectively. The catholyte in cathode compartment was 30 wt % [BMIM][PF_6]/MeCN or [BMMIM][PF_6]/MeCN solution. In addition, 0.1 M aqueous H_2SO_4 solution was used as the anolyte in anode chamber. The two chambers were separated by a Nafion 117 proton exchange membrane. Before reaction, the cathode chamber was injected with CO_2 at the flow rate of 30 mL/min for 30 min to saturate the electrolyte with CO_2, and continuously injected at the same rate during the electrolysis.

The gaseous products were collected by a gas bag and then directly analyzed via GC-MS, and the liquid product (HCOOH) was identified by ^{1}H NMR in *d*6-DMSO with TMS (0.03 %, v/v) as an internal standard.[(2)] The Faradaic efficiency (FE) for CO, HCOOH and H_2 was calculated according to the following equation:[(3, 4)]

$$FE_{(CO,H_2\ or\ HCOOH)} = \frac{n_{(CO,H_2\ or\ HCOOH)} F N_{(CO,H_2\ or\ HCOOH)}}{Q} \quad (1)$$

where n is the number of charge transfers (here n = 2), F is the Faraday constant (96485 C mol^{-1}), N is the molar mass of the corresponding product, Q is the total charge in the electrolysis that can be directly calculated from the i-t curve.

The electrochemically active surface (ECSA) of catalysts is proportional to the

double-layer capacitance (C_{dl}) determined from cyclic voltammetry (CV) method at the scan rate of 2, 4, 6, 8, 20, 30, 50, and 100 mV/s, respectively. The C_{dl} was quantified from the slope of the linear correlation between the current density differences ($\Delta j/2 = (j_a - j_c)/2$) at the midpoint of the CV potential window and the corresponding scan rate.[5]

DFT calculations

The density functional theory (DFT) calculations on geometrical structures and energies are performed by using the Vienna ab initio Simulation Package (VASP) at the generalized gradient approximation (GGA)/Perdew-Burke-Ernzerhof (PBE) level.[6, 7] The cut-off energy was set to 450 eV, while the wavefunctions were self-consistently optimized to within 10^{-5} eV using a 3×3×1 k-point mesh grid.[8] All atomic positions were allowed to relax until the forces were less than 0.02 eV/Å. Electron smearing of σ=0.05 eV was used following the Gaussian scheme. To ensure that the calculation results are reasonable, dispersion-corrected DFT-D3 method[9] was set for absorption models. The calculation data were analyzed by vaspkit Code.[10]

The adsorption energies on these model systems were calculated using the following equation:

$$E_{ads} = E_{total} - E_{structure} \tag{2}$$

where E_{ads} represents the adsorption energy, E_{total} is the total energy of the optimized structure including the adsorbate, and $E_{structure}$ is the energy of the optimized structure without the adsorbate, plus the energy of each individual adsorbate's optimized structure.

2. Figures S1-S29

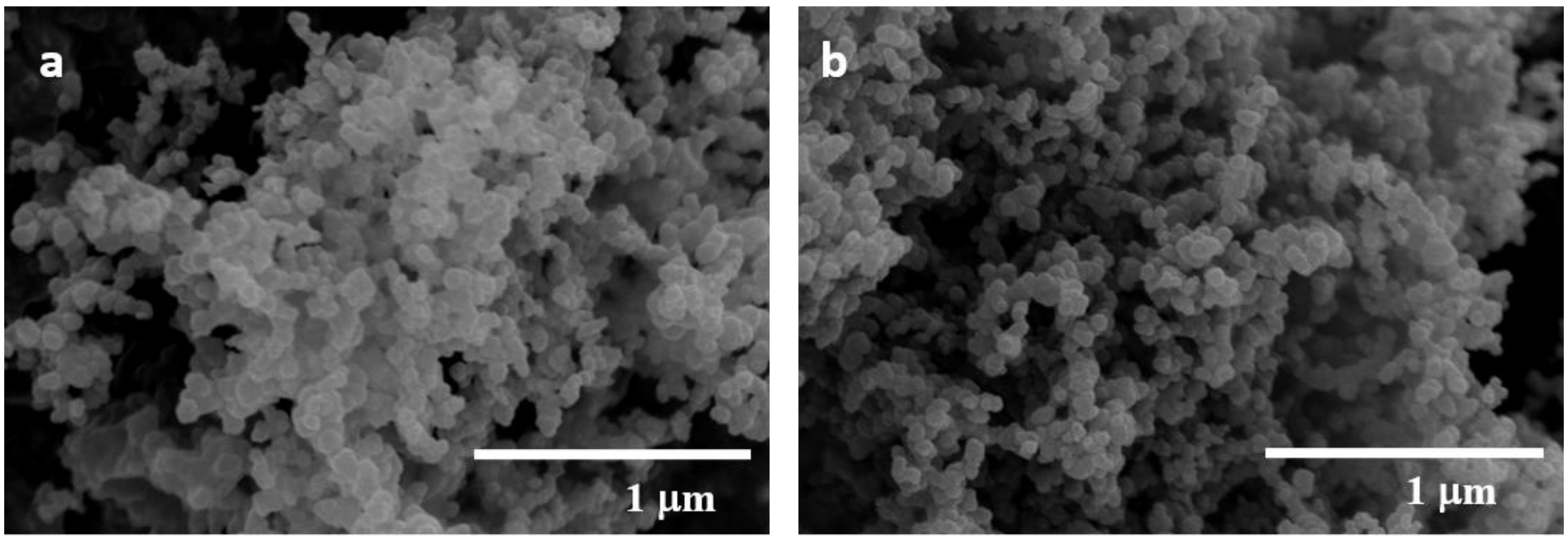

Figure S1. SEM images of (a) Fe_2Sn_1NC and (b) $Fe_{58}Sn_1NC$.

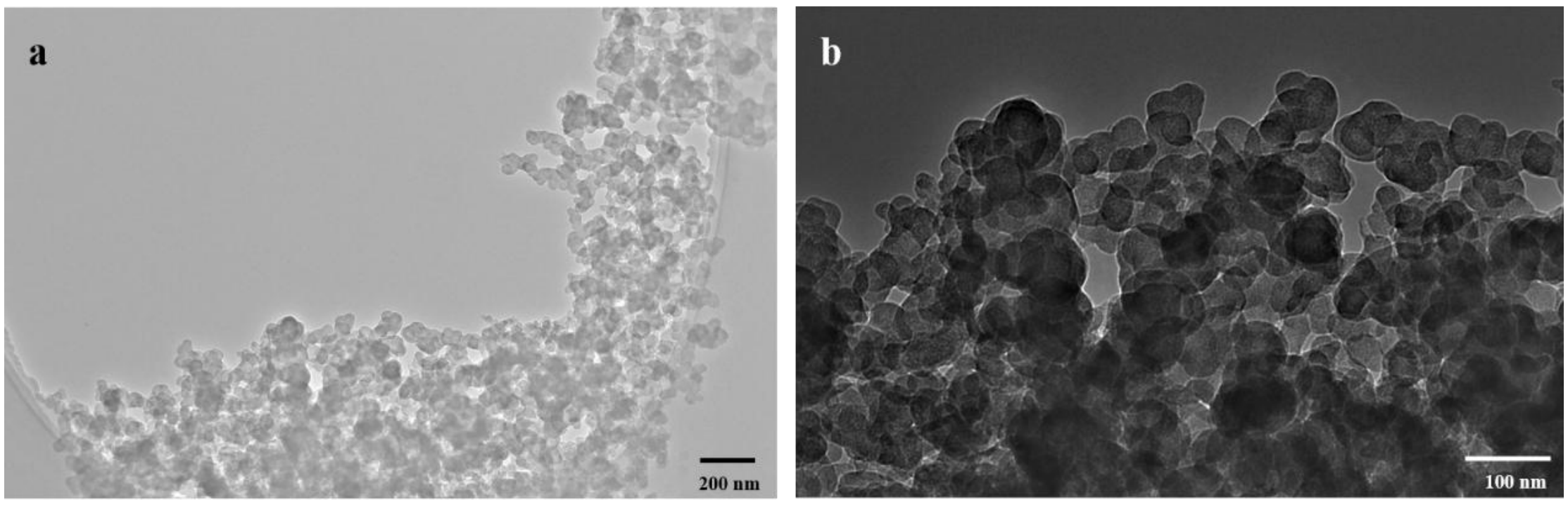

Figure S2. TEM images of Fe_5Sn_1NC under the scale bar of (a) 200 nm and (b) 100 nm.

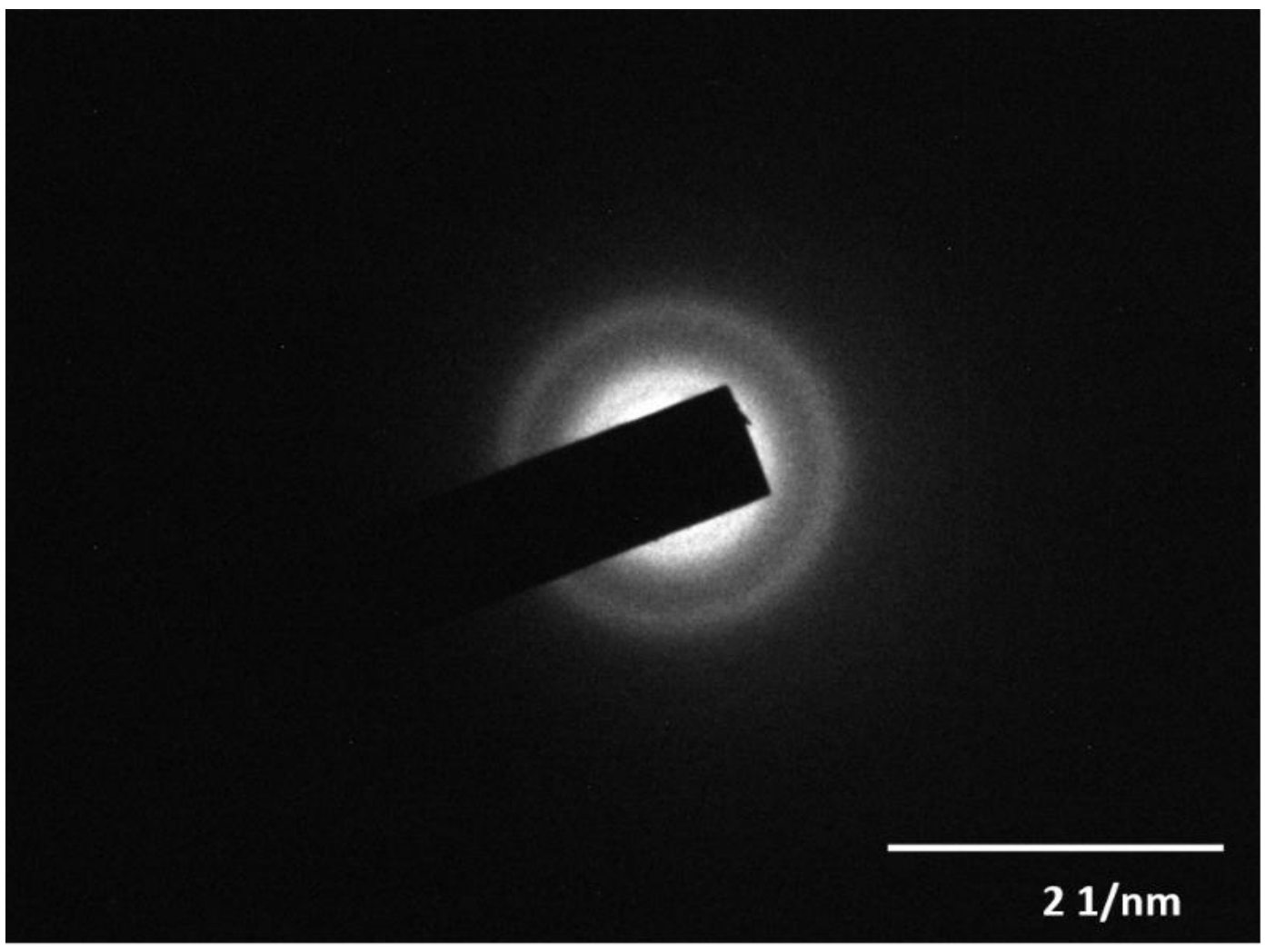

Figure S3. SAED pattern of Fe_5Sn_1NC.

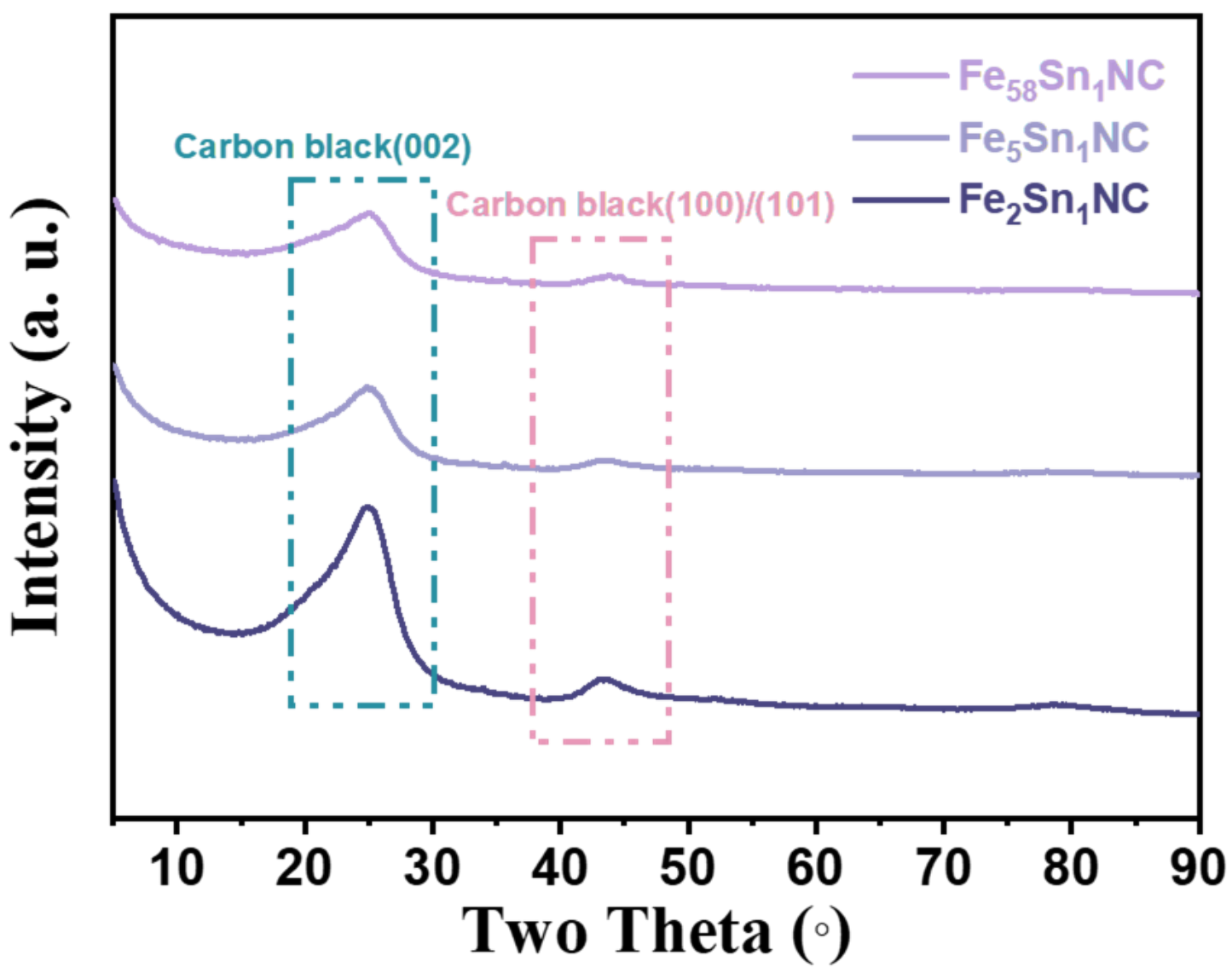

Figure S4. XRD pattern of Fe_2Sn_1NC, Fe_5Sn_1NC and $Fe_{58}Sn_1NC$.

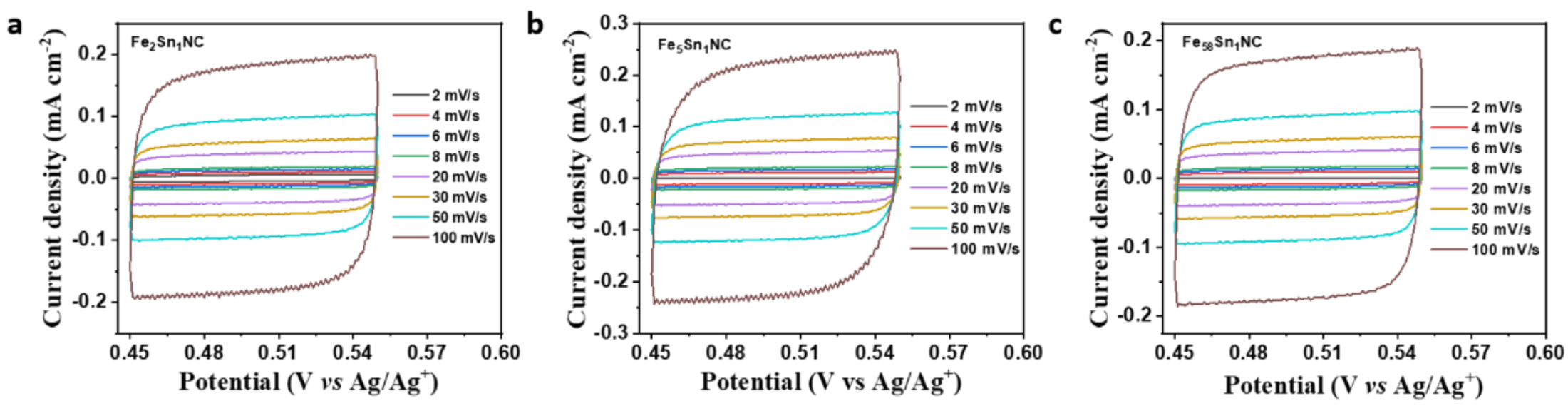

Figure S5. Cyclic voltammograms at the potential range from -0.45 to -0.55 V (*vs* Ag/Ag^+) with the scan rate of 2, 4, 6, 8, 20, 30, 50, and 100 mV/s, respectively, for (a) Fe_2Sn_1NC, (b) Fe_5Sn_1NC, and (c) $Fe_{58}Sn_1NC$ in 30 wt % [BMMIM][PF_6]/MeCN.

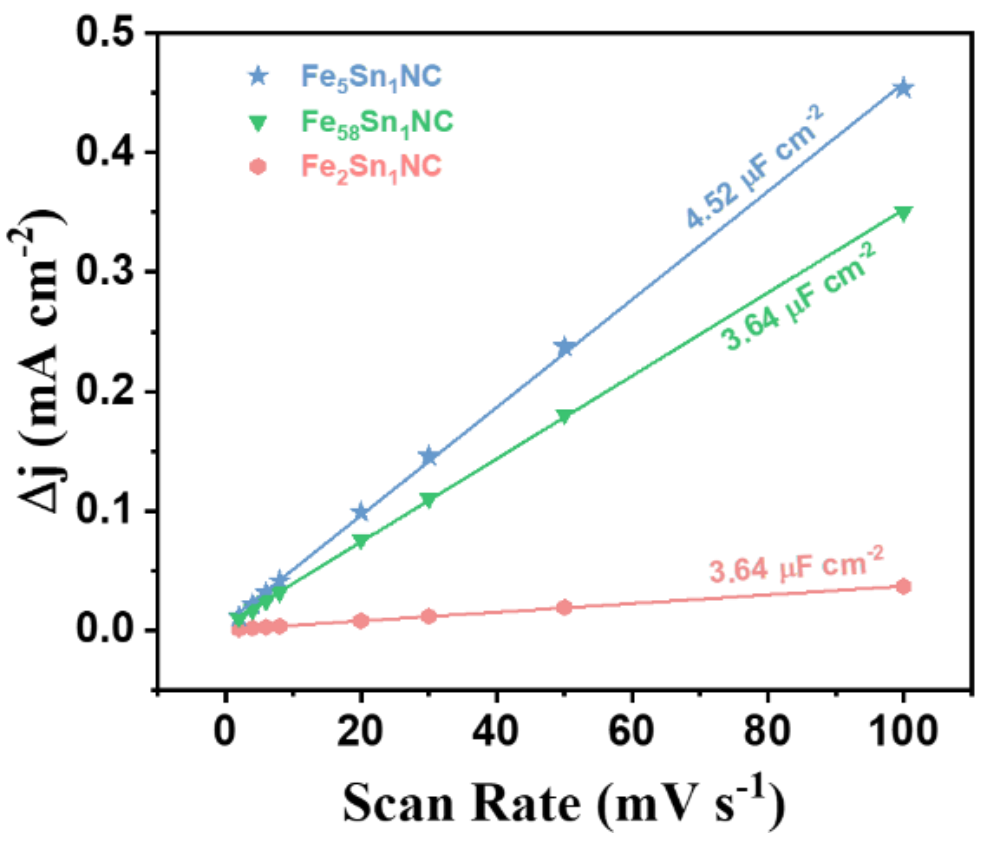

Figure S6. Electrochemical double layer capacitance of Fe_2Sn_1NC, Fe_5Sn_1NC and $Fe_{58}Sn_1NC$ in 30 wt % [BMMIM][PF_6]/MeCN.

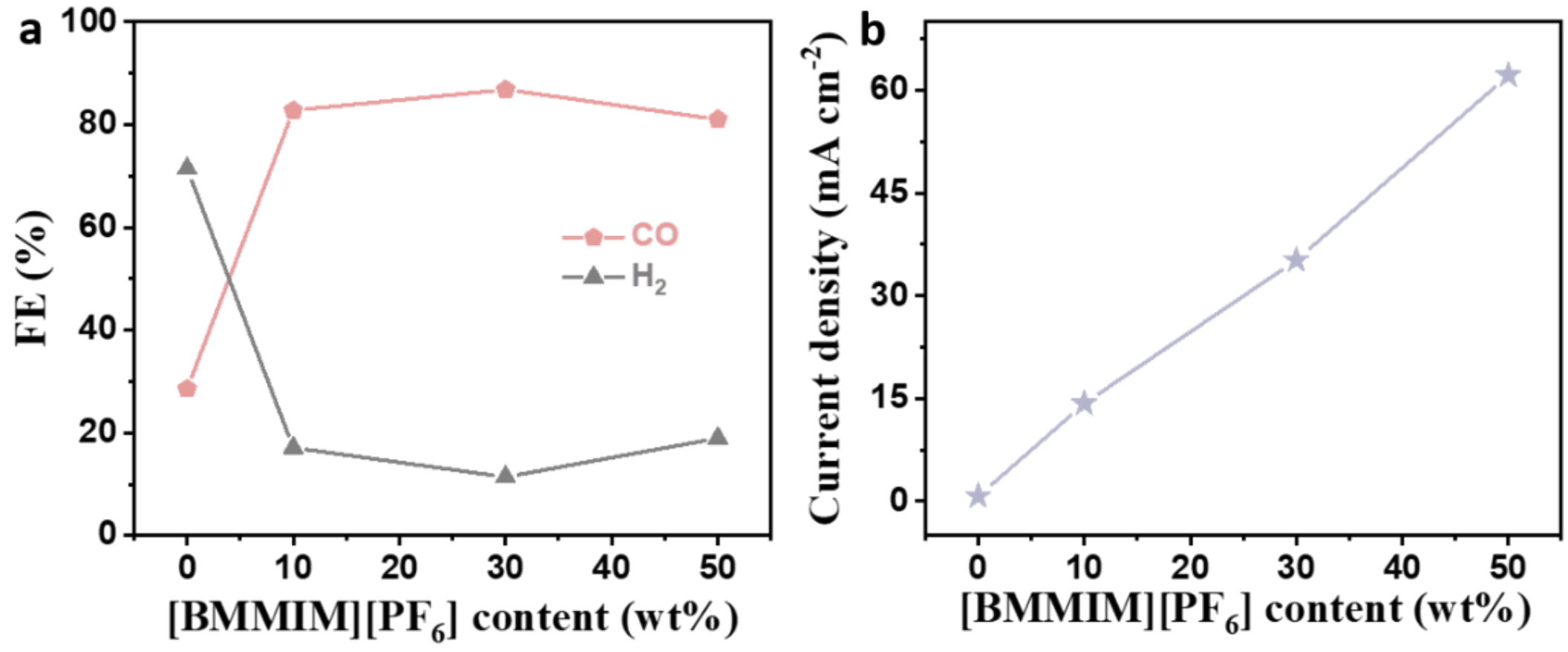

Figure S7. Optimization of [BMMIM][PF_6] content in the electrolytes: (a) FE of CO and H_2, and (b) total current density from CO_2RR under the catalysis of Fe_5Sn_1NC.

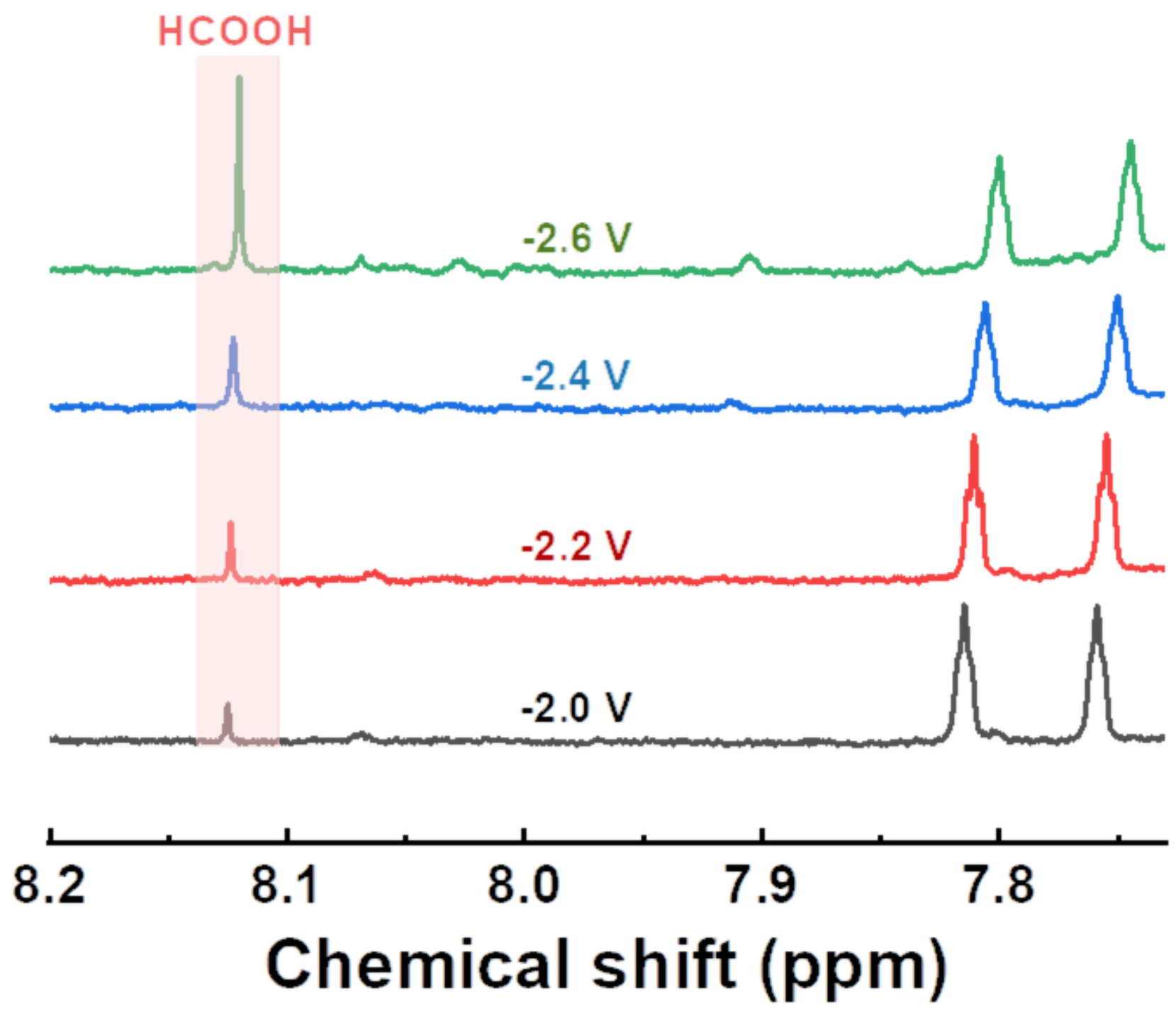

Figure S8. ^{1}H NMR spectroscopy verification of HCOOH produced from CO_2RR in 30 wt % [BMIM][PF_6]/MeCN under the catalysis of Fe_5Sn_1NC.

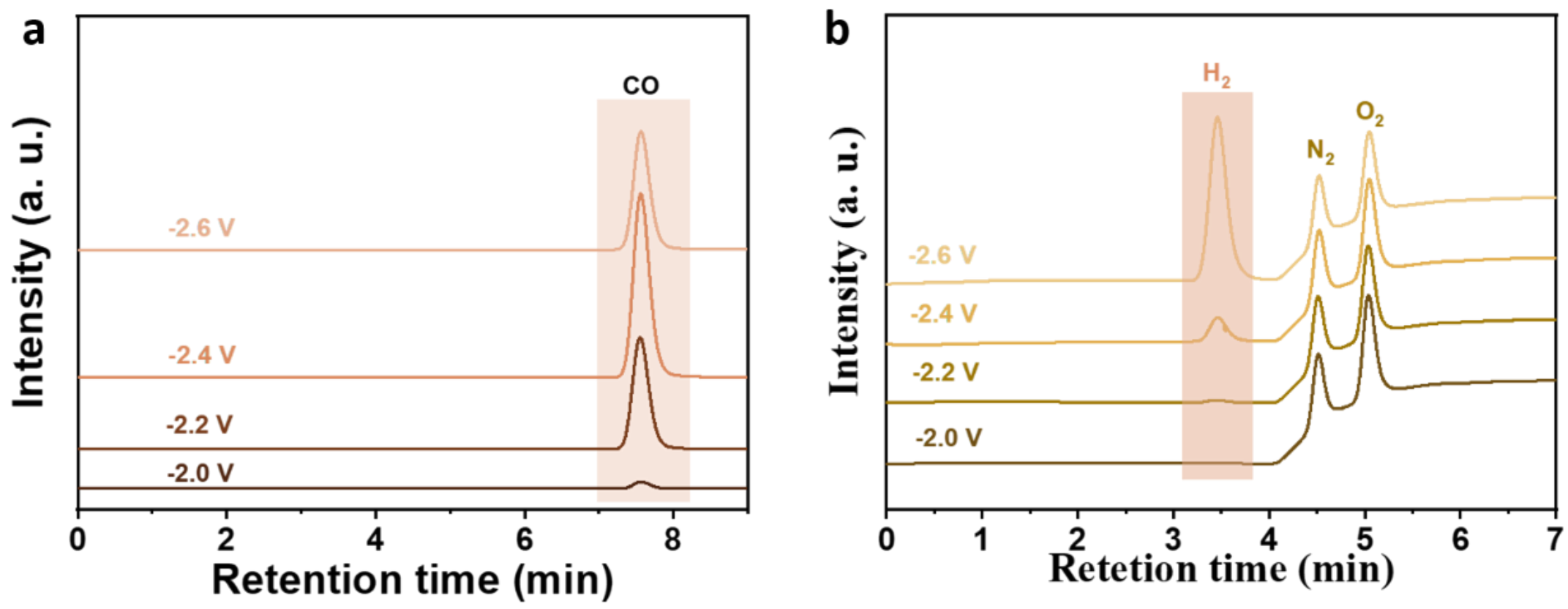

Figure S9. Gas chromatography verification of CO (a) and H_2 (b) produced from CO_2RR in 30 wt % [BMMIM][PF_6]/MeCN under the catalysis of Fe_5Sn_1NC.

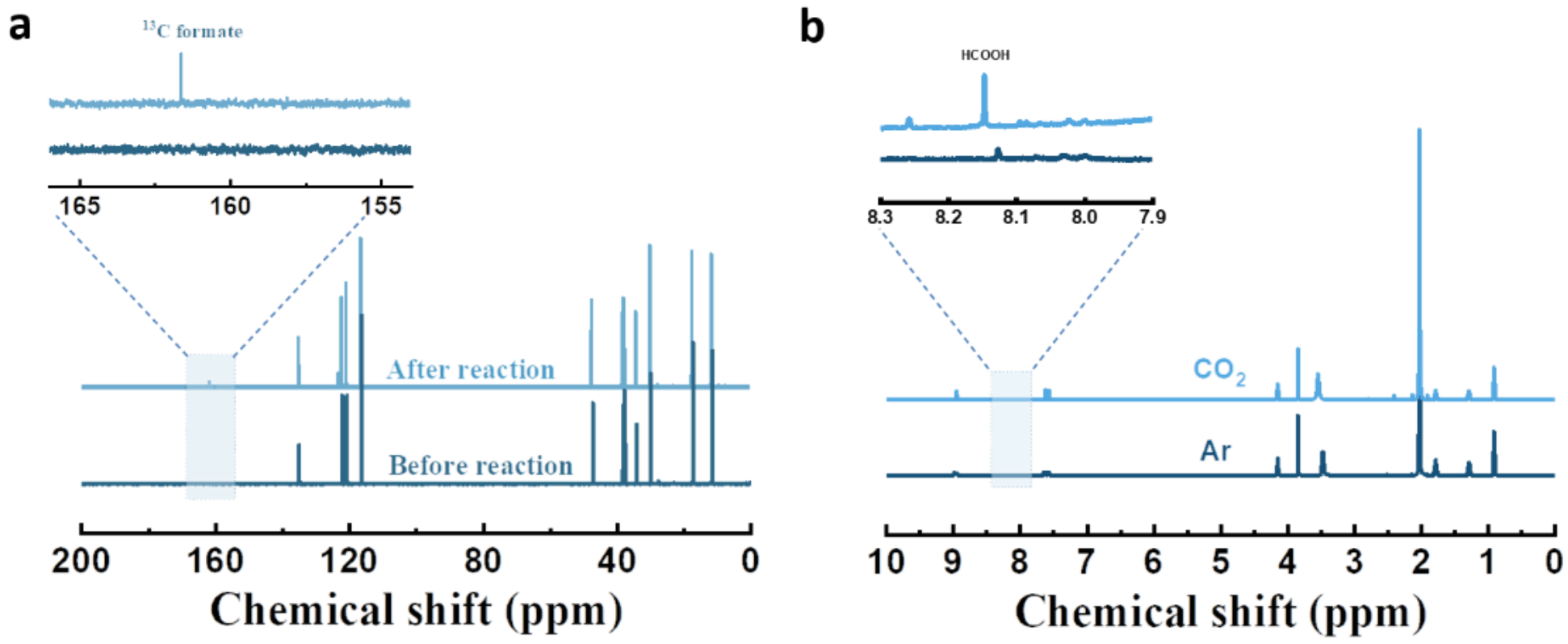

Figure S10. The verification of carbon source for HCOOH by using (a) ^{13}C NMR with labeled $^{13}CO_2$ and (b) ^{1}H NMR for Ar-saturated instead of CO_2-saturated CO_2RR in 30 wt % [BMIM][PF_6]/MeCN under the catalysis of Fe_5Sn_1NC at -2.6 V (*vs* Ag/Ag^+).

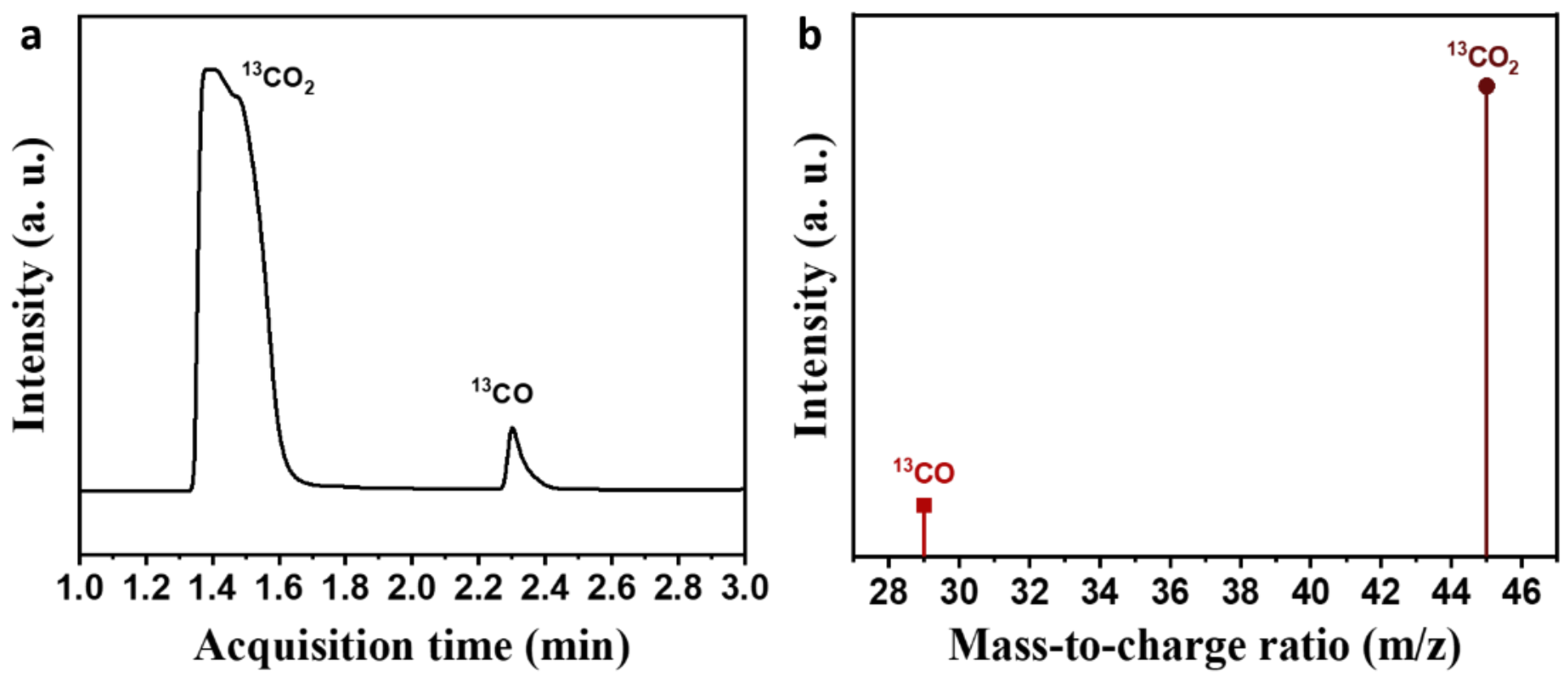

Figure S11. Gas chromatography verification of carbon source for CO by using labeled $^{13}CO_2$ to perform CO_2RR in 30 wt % [BMMIM][PF_6]/MeCN under the catalysis of Fe_5Sn_1NC at -2.2 V (*vs* Ag/Ag^+).

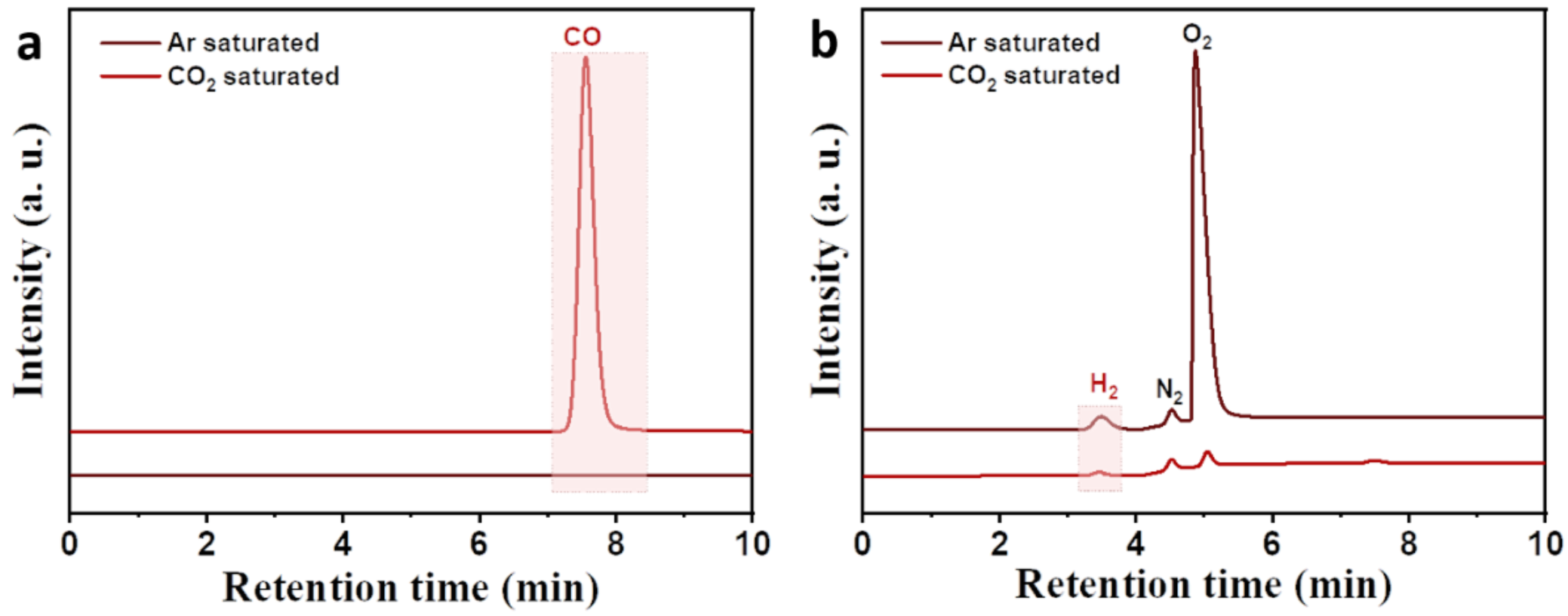

Figure S12. Gas chromatography verification of carbon source for CO by using Ar-saturated instead of CO_2-saturated CO_2RR in 30 wt % [BMMIM][PF_6]/MeCN under the catalysis of Fe_5Sn_1NC at -2.2 V (*vs* Ag/Ag^+).

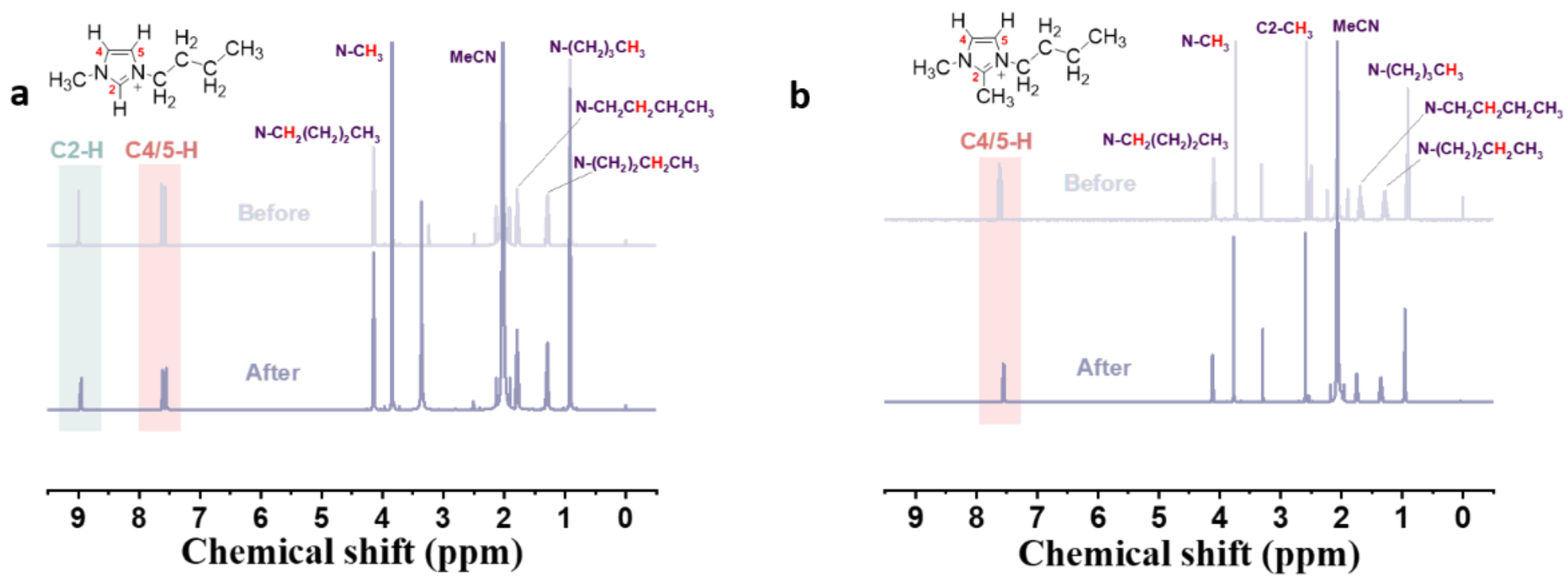

Figure S13. ^{1}H-NMR spectra of (a) 30 wt % [BMIM][PF_6]/MeCN before and after the electrolysis at -2.6 V (*vs* Ag/Ag^+) and (b) 30 wt % [BMMIM][PF_6]/MeCN before and after the electrolysis at -2.2 V (*vs* Ag/Ag^+).

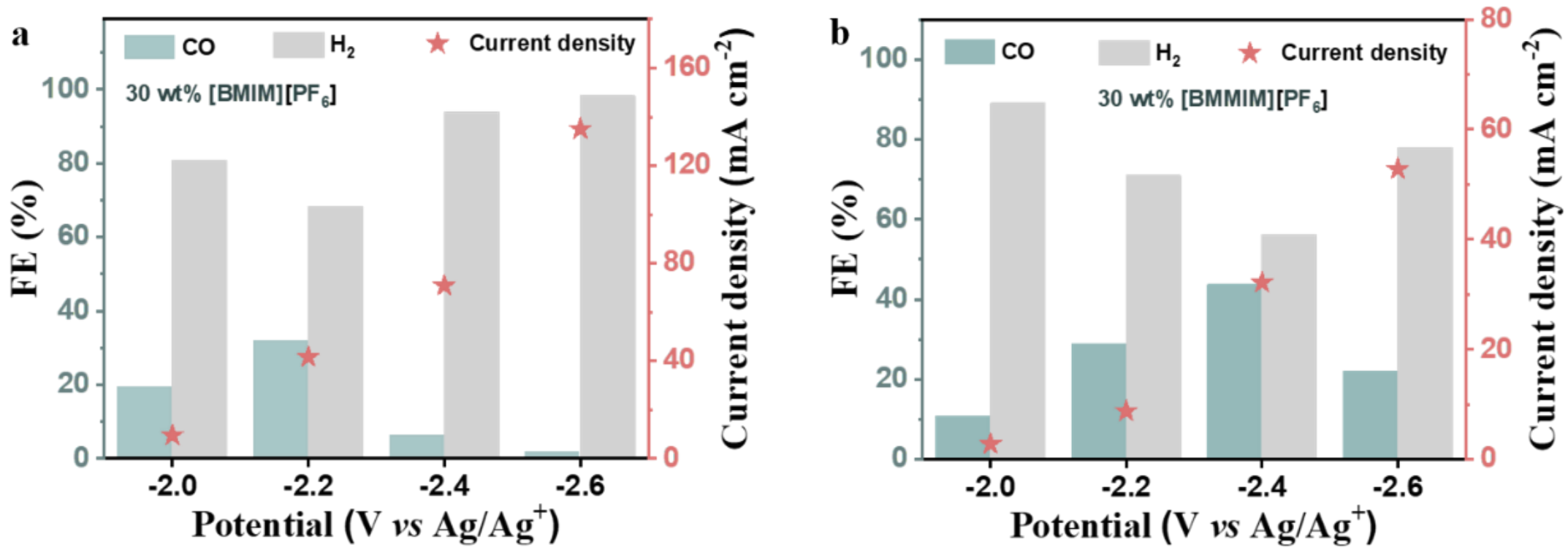

Figure S14. CO_2RR performance with the carbon black as the catalyst in (a) 30 wt % [BMIM][PF_6]/MeCN and (b) 30 wt % [BMMIM][PF_6]/MeCN.

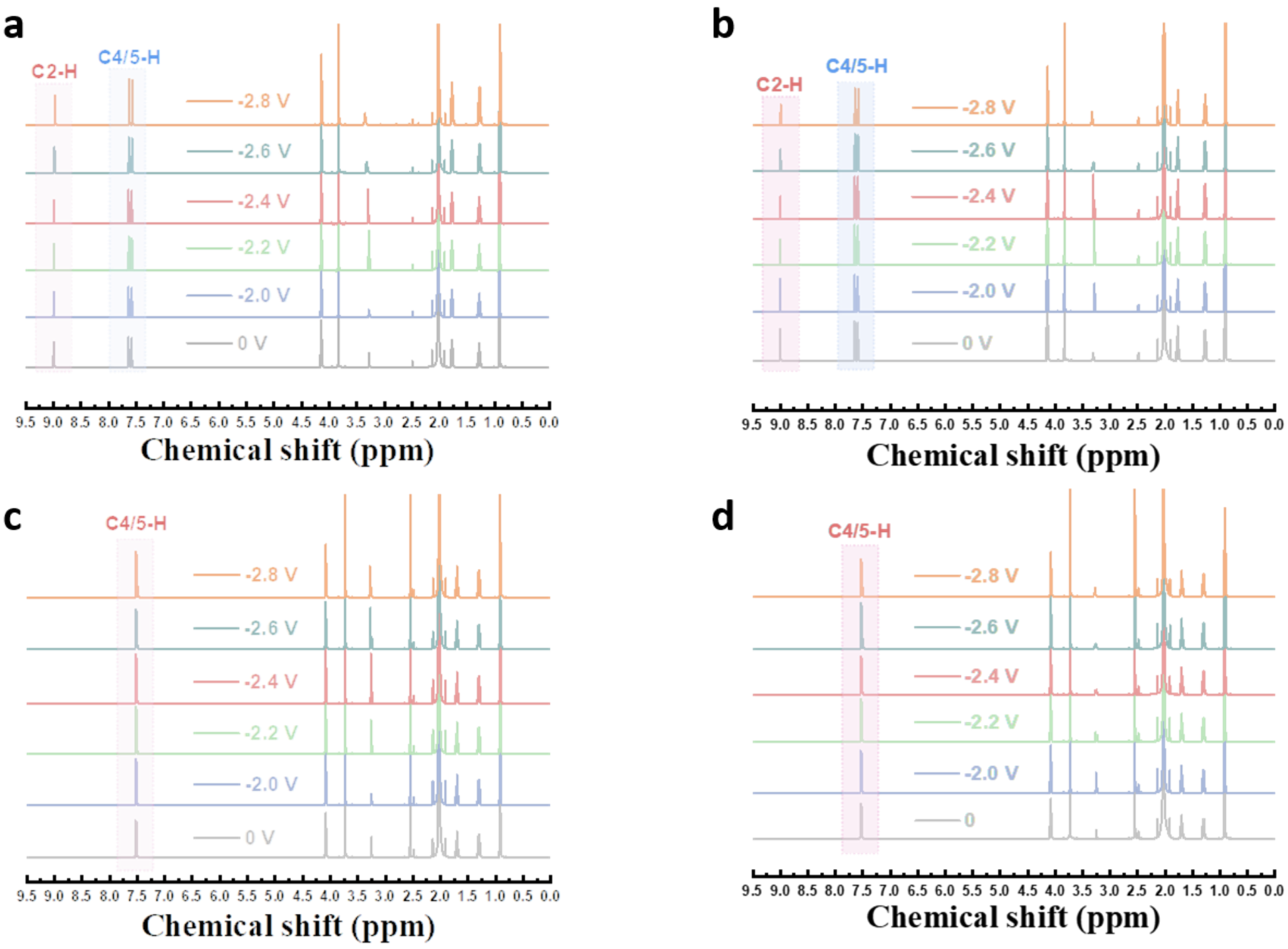

Figure S15. ^{1}H NMR spectra of the electrolytes: (a) 30 wt % [BMIM][PF_6]/MeCN before and after electrolysis (-2.0 to -2.8 V, *vs* Ag/Ag^+) under Ar atmosphere. (b) 30 wt % [BMIM][PF_6]/MeCN before and after electrolysis (-2.0 to -2.8 V, *vs* Ag/Ag^+) under CO_2 atmosphere. (c) 30 wt % [BMMIM][PF_6]/MeCN before and after electrolysis (-2.0 to -2.8 V, *vs* Ag/Ag^+) under Ar atmosphere. (d) 30 wt % [BMMIM][PF_6]/MeCN before and after electrolysis (-2.0 to -2.8 V, *vs* Ag/Ag^+) under CO_2 atmosphere.

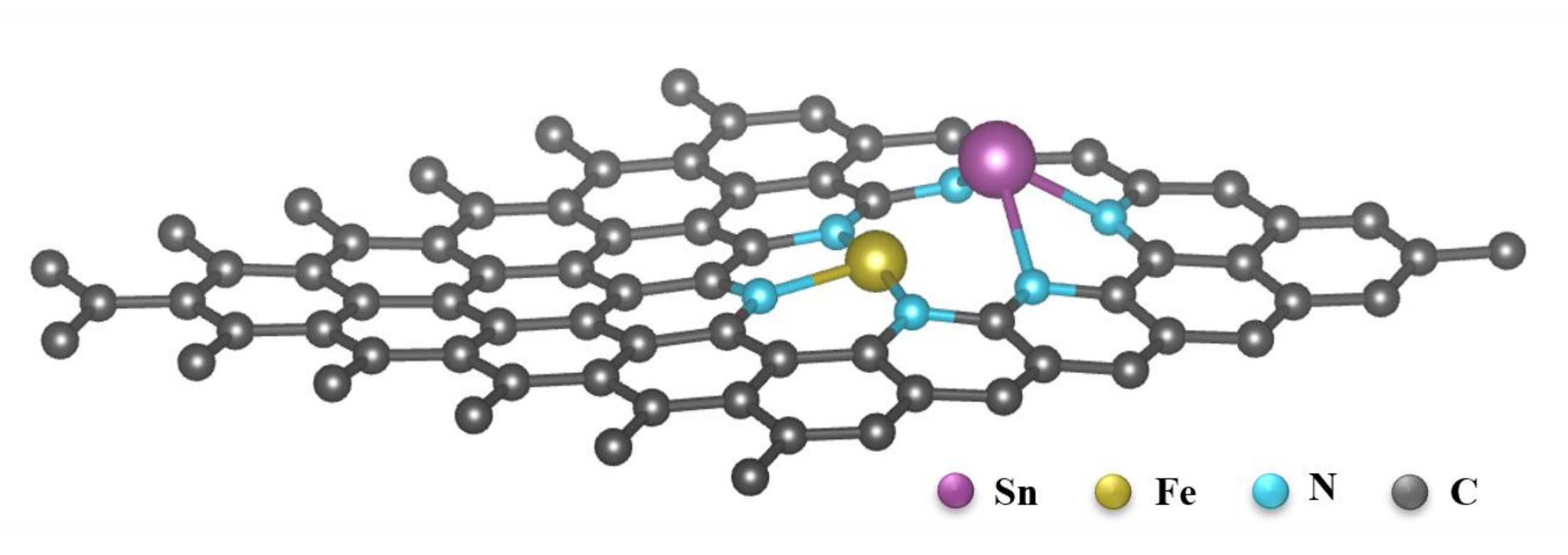

Figure S16. The optimized structure of Fe_5Sn_1NC catalyst.

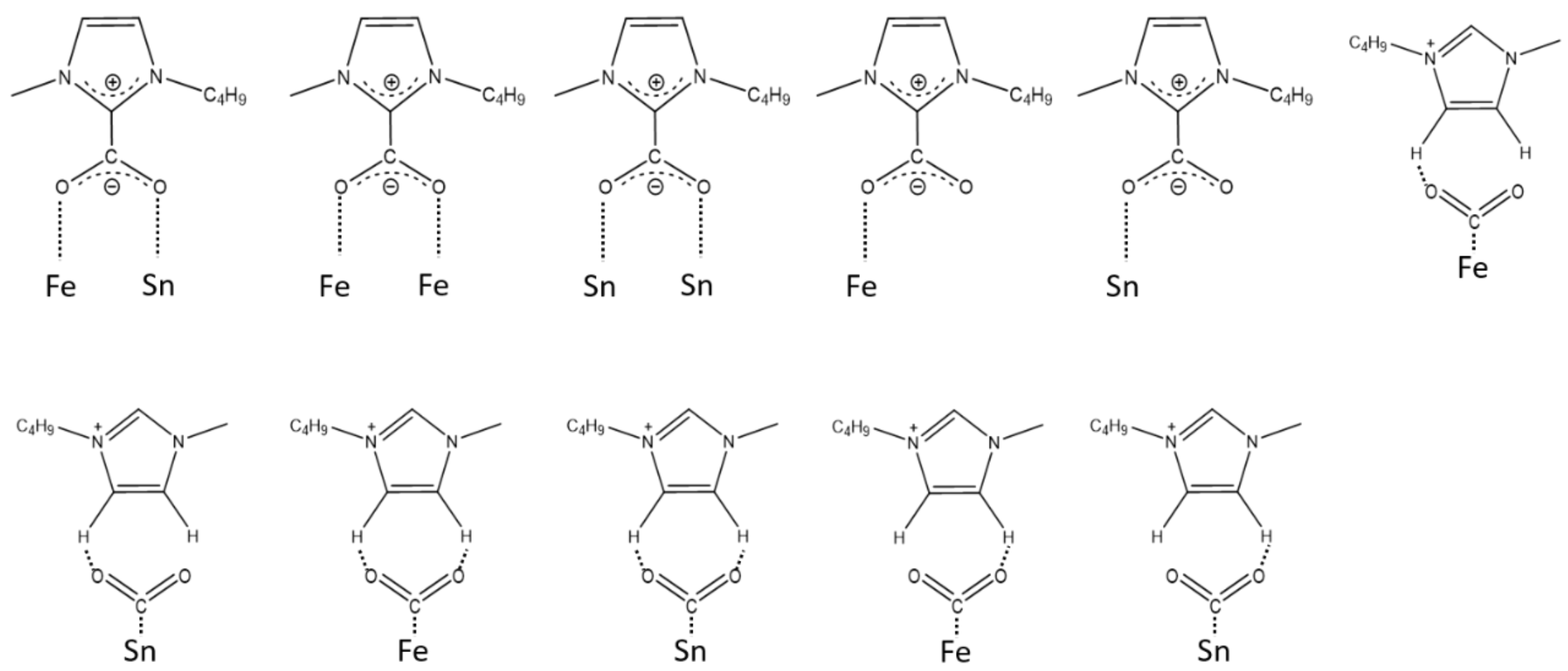

Figure S17. All the possible CO_2 adsorption configurations on the Fe_5Sn_1NC catalyst surface in the $[BMIM]^+$-based system.

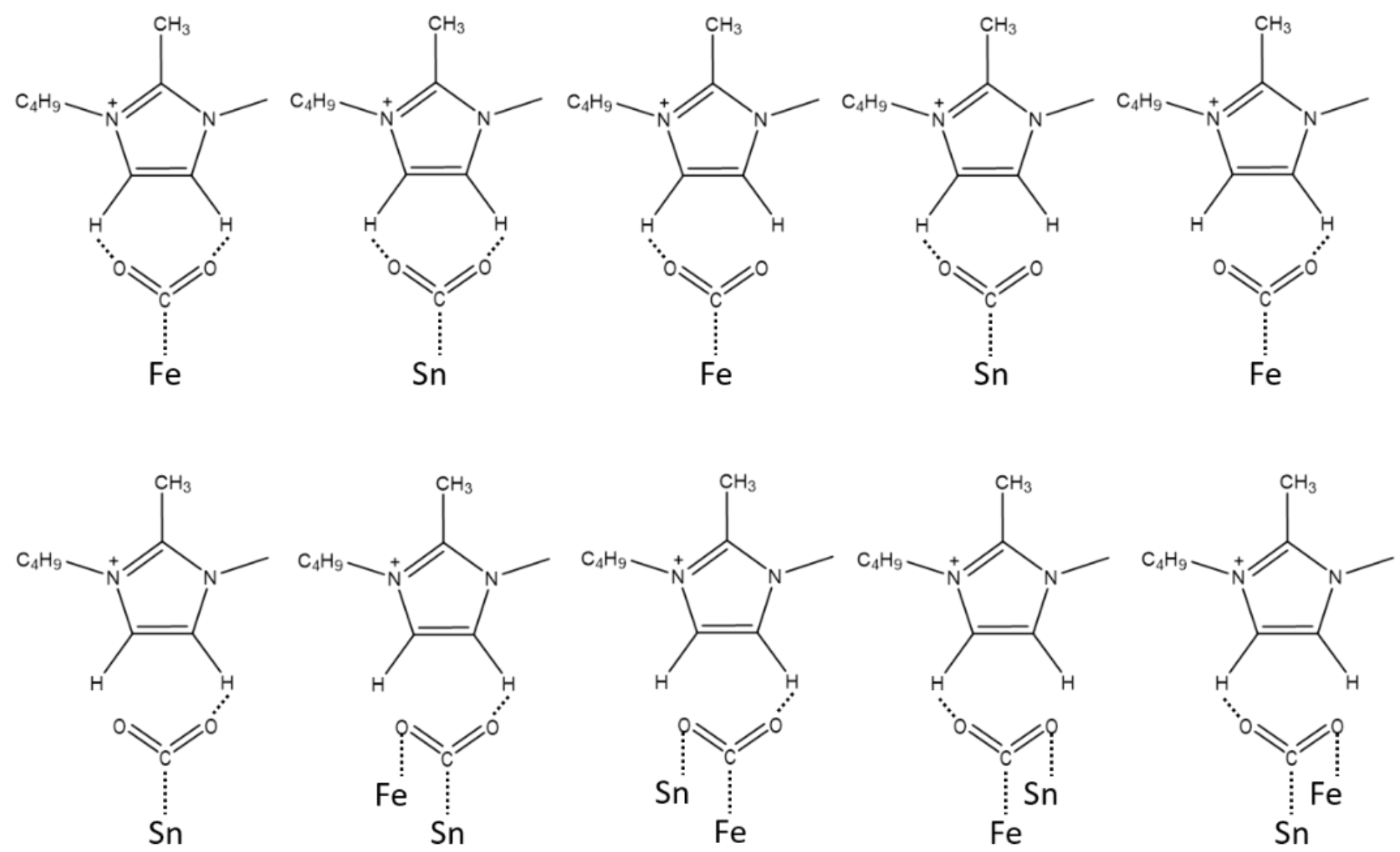

Figure S18. All the possible CO_2 adsorption configurations on the Fe_5Sn_1NC catalyst surface in the $[BMMIM]^+$-based system.

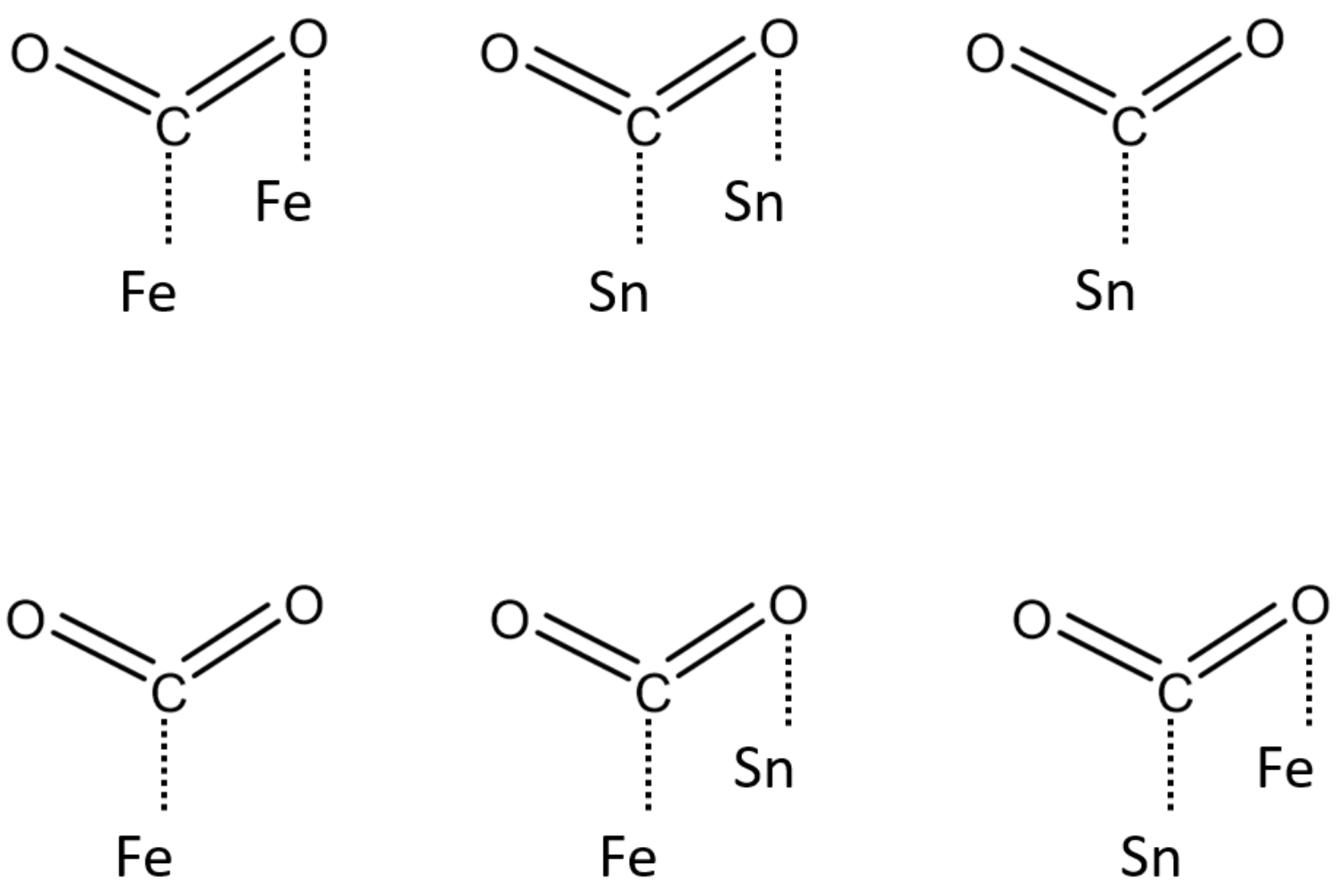

Figure S19. All the possible CO_2 adsorption configurations on the Fe_5Sn_1NC catalyst surface without ILs.

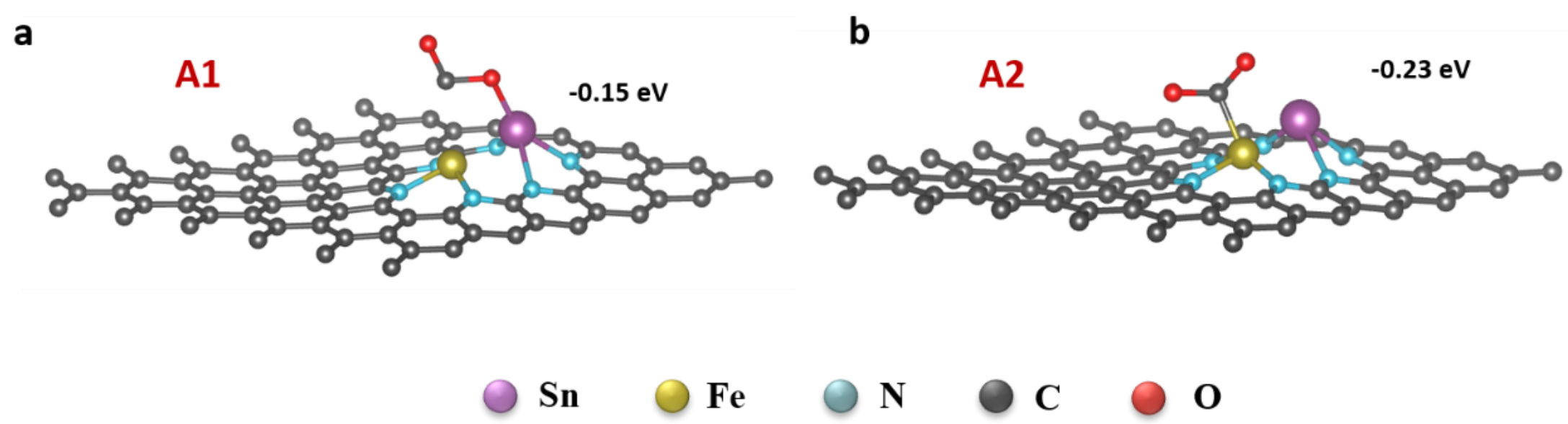

Figure S20. The optimized CO_2 adsorption configuration on the catalyst surface and the corresponding adsorption energy in the absence of ILs.

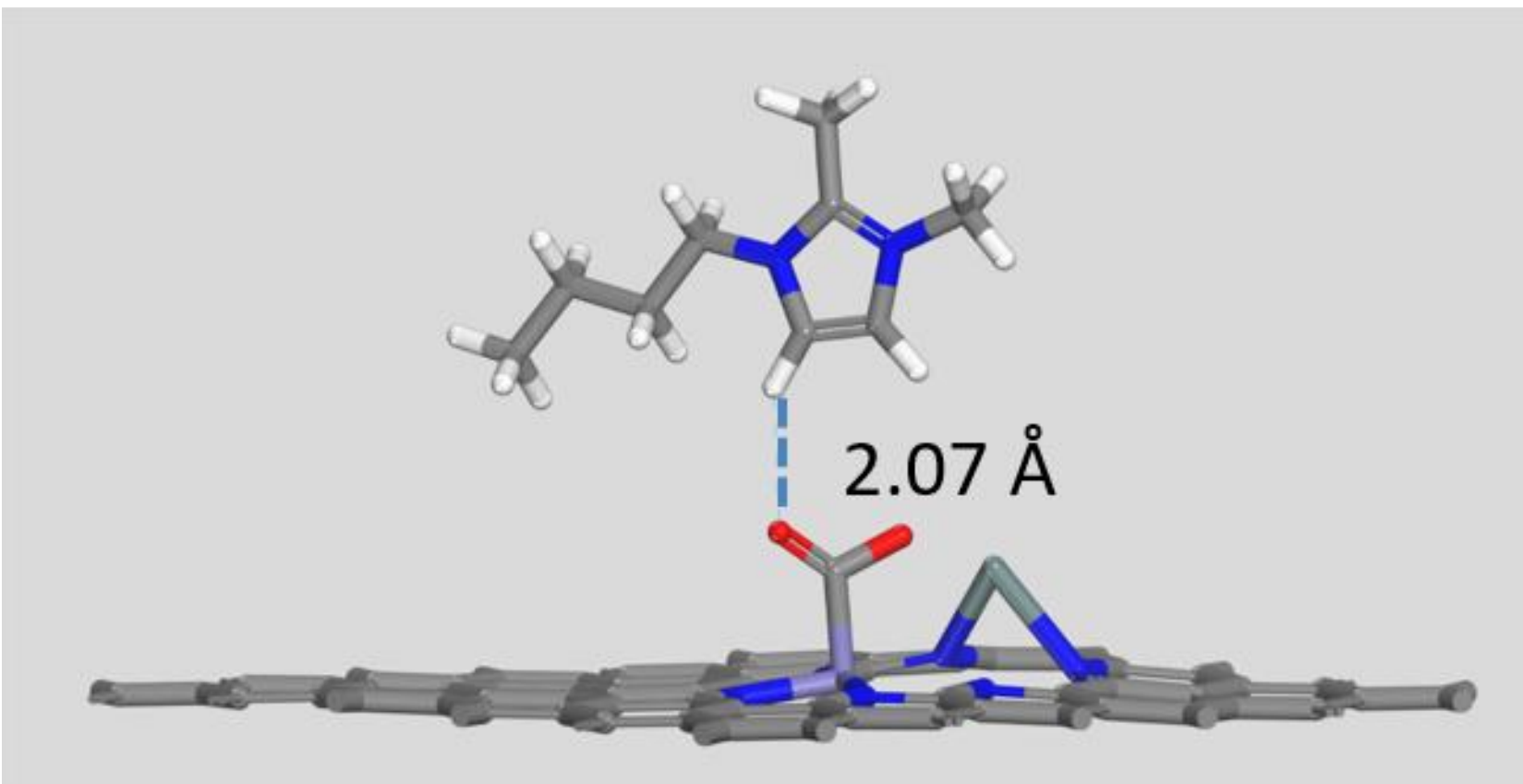

Figure S21. The optimized CO_2 adsorption configuration on the catalyst surface in the $[BMMIM]^+$-based electrolyte. Broken line shows the hydrogen bonding interaction.

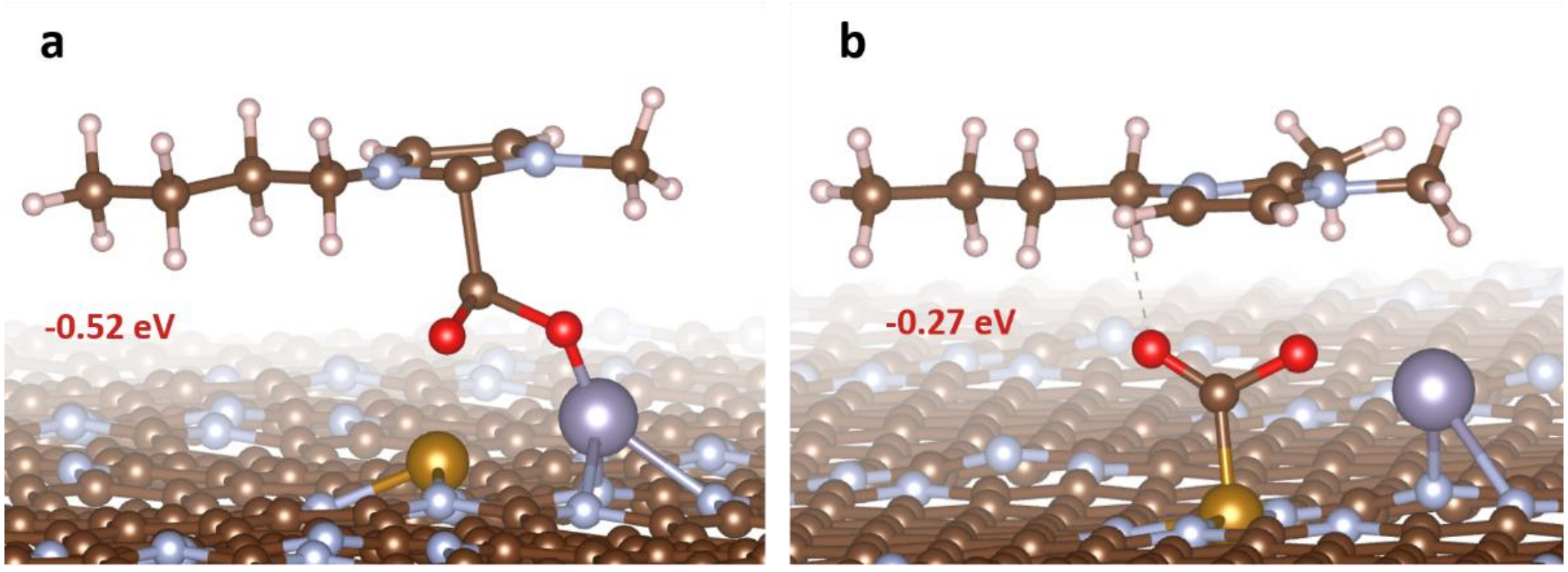

Figure S22. CO_2 adsorption configurations on the catalyst surface and the corresponding adsorption energy of CO_2 on the catalyst in the presence of a parallel-oriented (a) [BMIM]$^+$ and (b) [BMMIM]$^+$ relative to the catalyst surface.

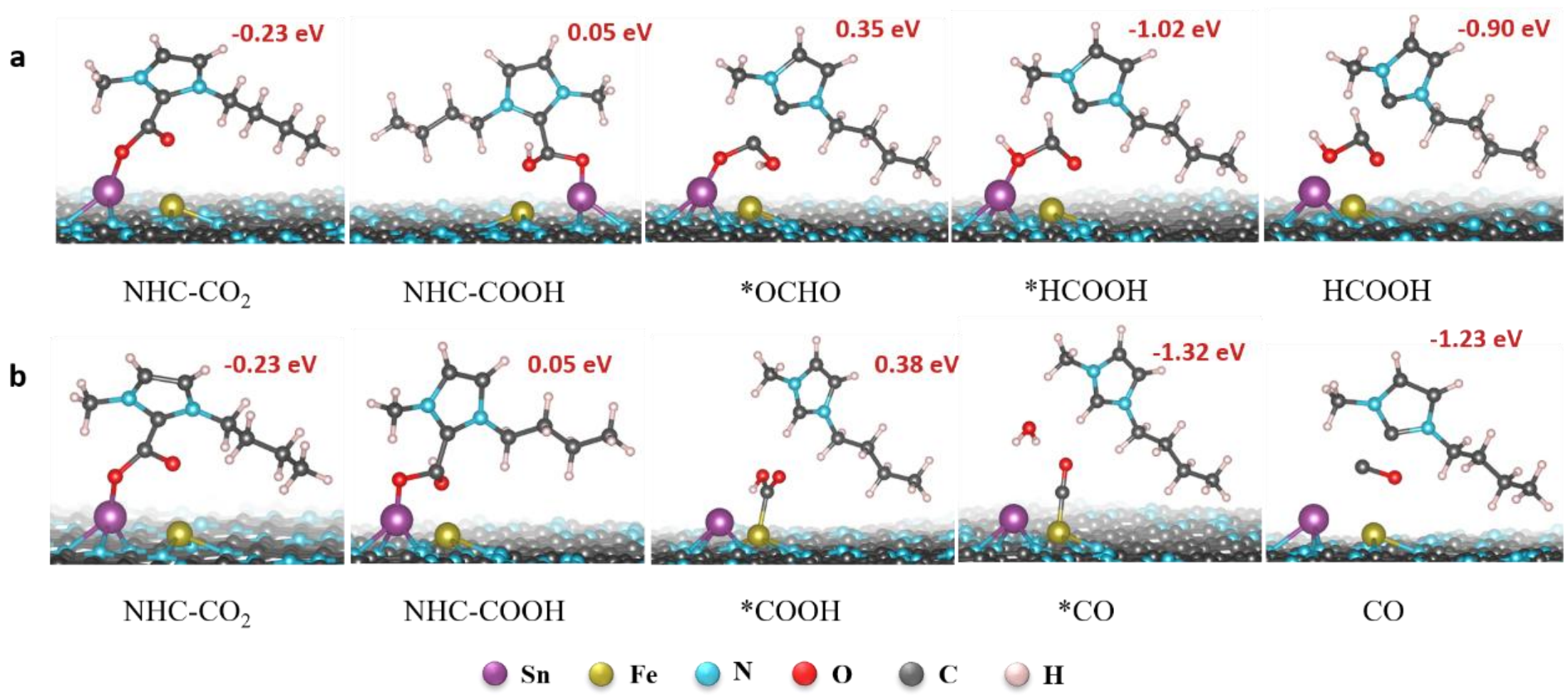

Figure S23. The optimized adsorption configurations of the reaction intermediates on the Fe_5Sn_1NC catalyst surface for the reaction pathway to CO and HCOOH, respectively, in [BMIM]$^+$-based electrolyte, and the corresponding adsorption free energy.

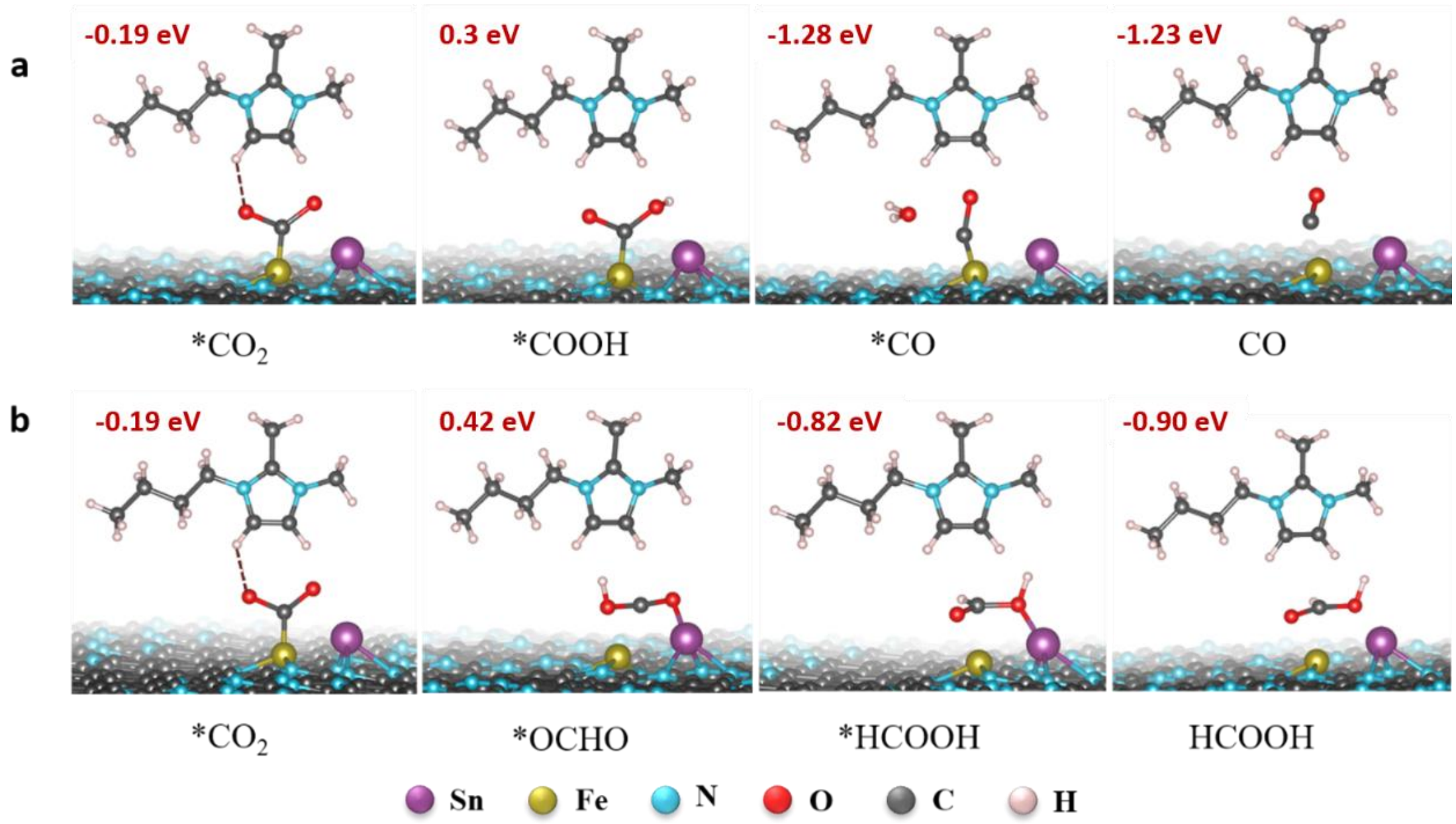

Figure S24. The optimized adsorption configurations of the reaction intermediates on the Fe_5Sn_1NC catalyst surface for the reaction pathway to CO and HCOOH, respectively, in $[BMMIM]^+$-based electrolyte, and the corresponding adsorption free energy.

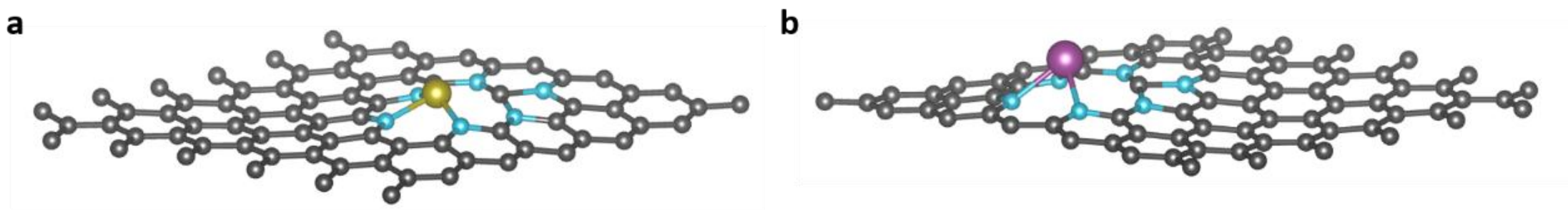

Figure S25. The optimized structure of FeNC (a) and SnNC (b) catalyst.

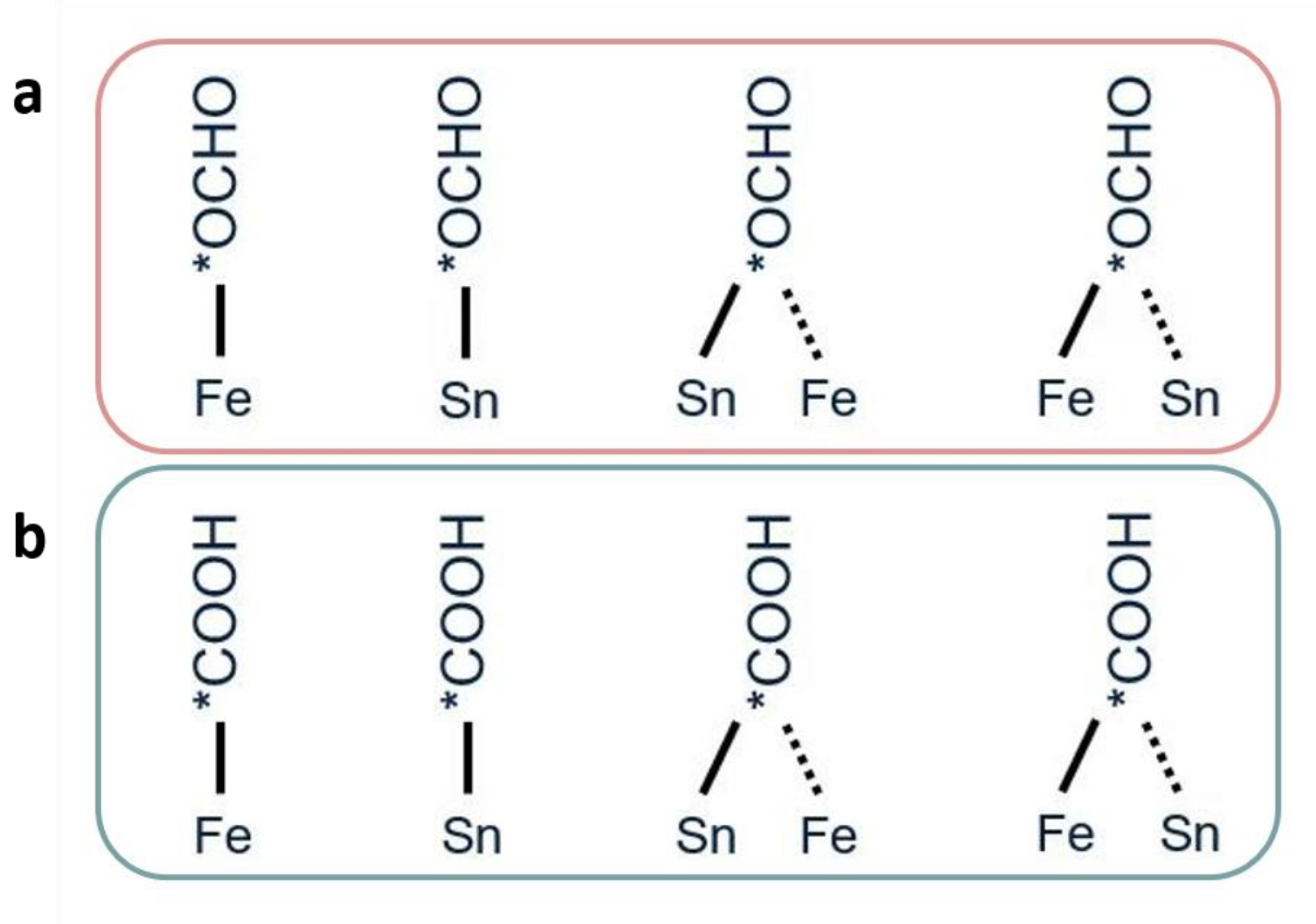

Figure S26. All the possible adsorption configurations of key intermediates on mono-/bimetallic catalyst surface in $[BMIM]^+$-based electrolyte (a) and $[BMMIM]^+$-based electrolyte (b).

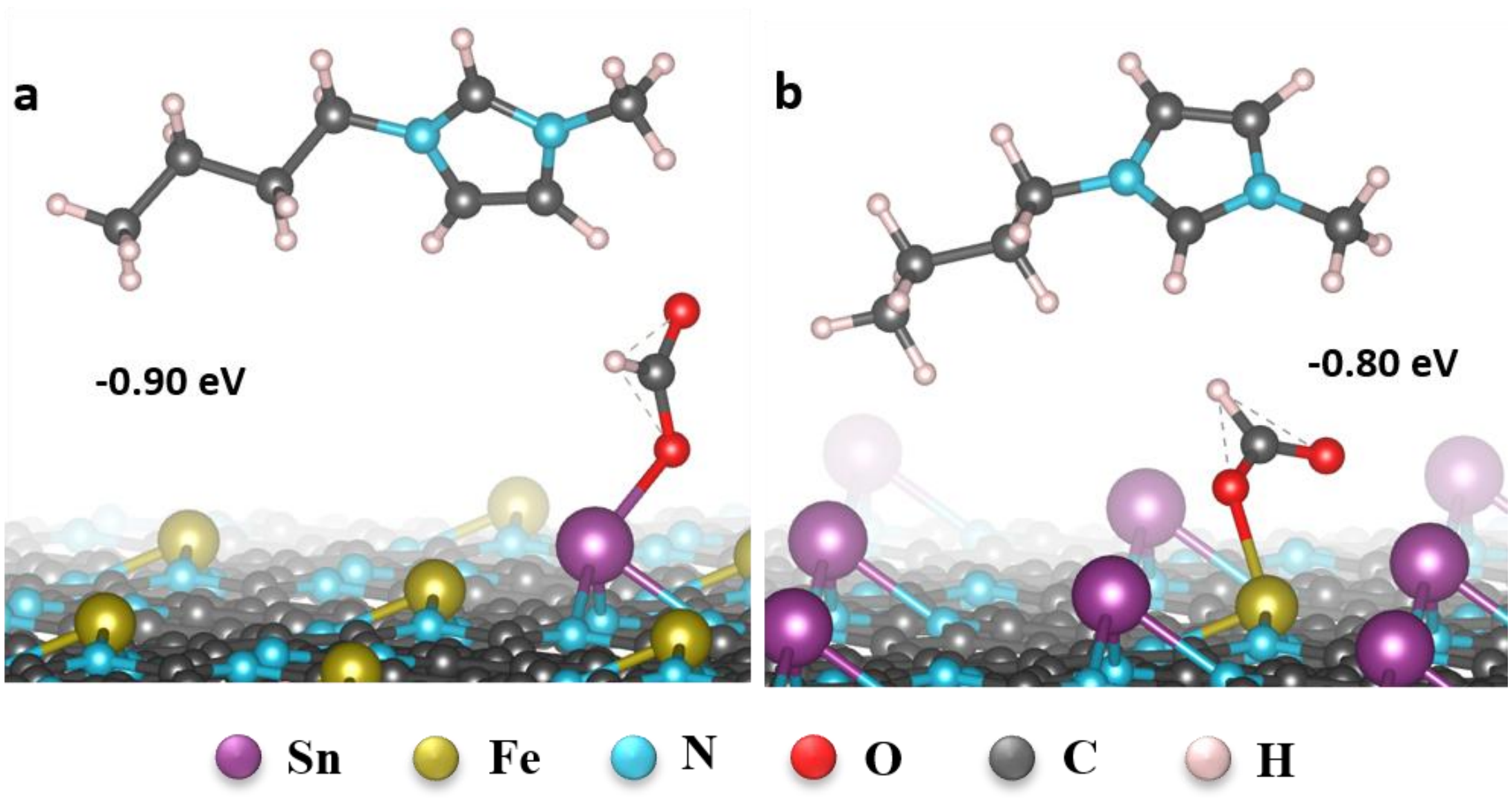

Figure S27. The optimized adsorption configurations of *OCHO in $[BMIM]^+$-based electrolyte and the corresponding adsorption energy.

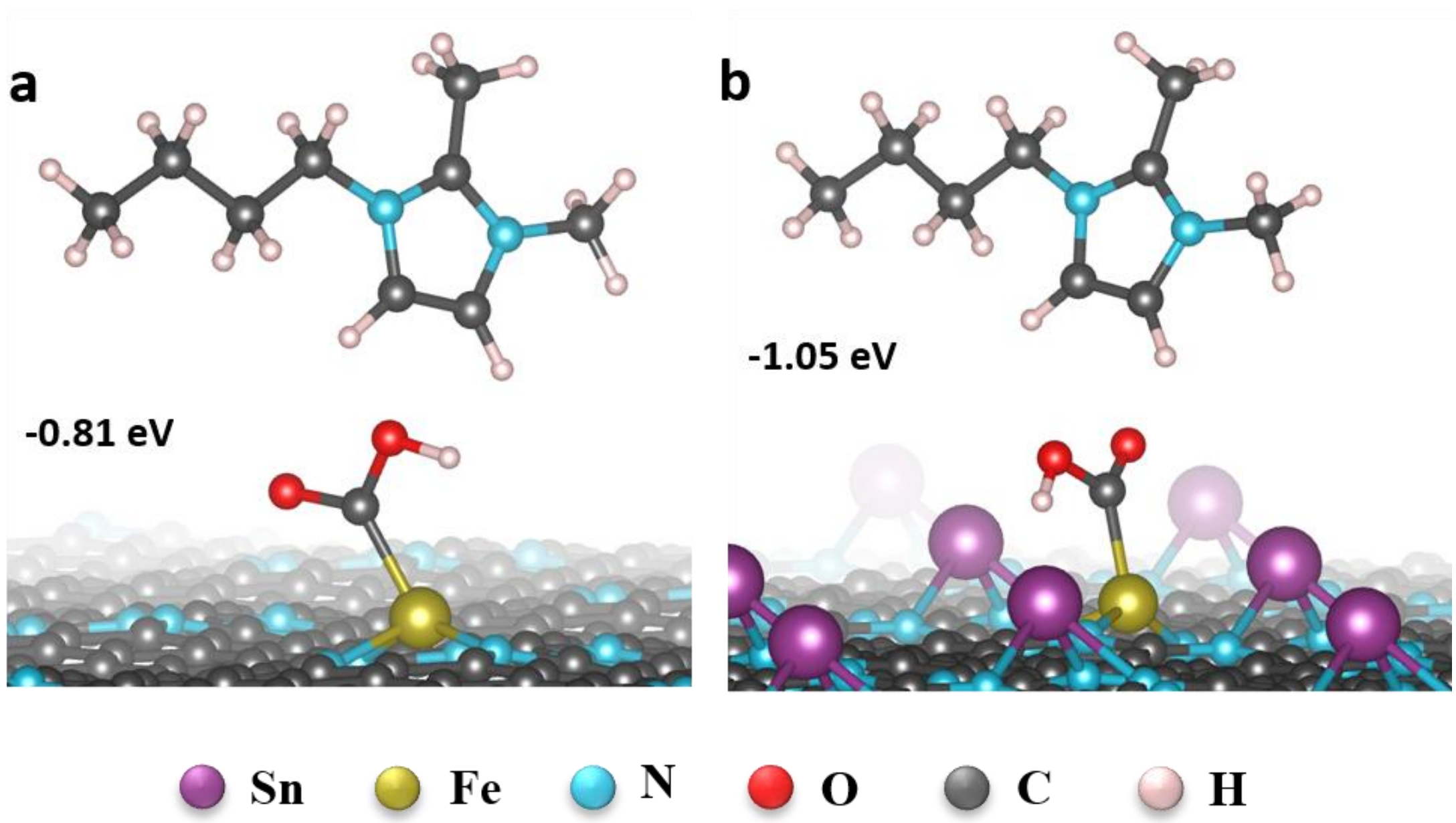

Figure S28. The optimized adsorption configurations of *COOH in [BMMIM]+-based electrolyte and the corresponding adsorption energy.

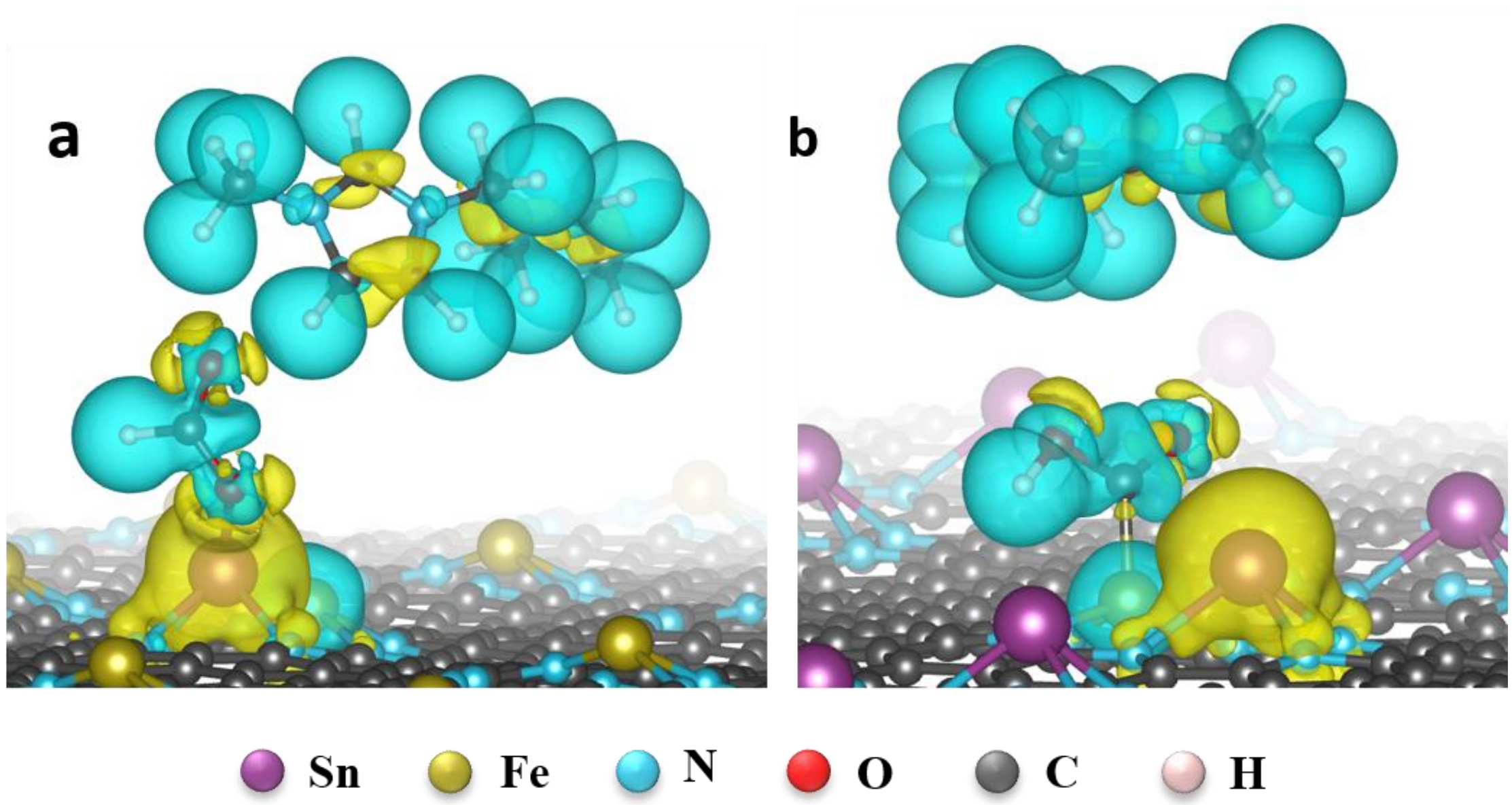

Figure S29. The charge density difference of Fe_5Sn_1NC with the adsorption of (a) *OCHO intermediate in [BMIM]+-based electrolyte and (b) *COOH intermediate in [BMMIM]+-based electrolyte.

3. Tables S1-S6

Table S1. Summary of CO_2RR-to-HCOOH performance in the IL-based electrolytes since 2021

Electrolyte	Catalyst	Potential (V)	FE_{HCOOH} (%)	Current density (mA/cm^2)	Electrolyzer	Ref.
250 mM [BMIM][PF_6]/ MeCN/H_2O	Cu	-1.5 V_{RHE}	52.79	/	H-cell	11
80 mmol L^{-1} H_2O /P_{1444}TFSI	Cu_2O	-0.9 V *vs* Fc/Fc$^+$	86	/	Single-compartment cell	12
5 vol.% H_2O /[EMIM][BF_4]/MeCN	[Rh(bpy)(Cp)*Cl]Cl	-1.83 V *vs* Fc/Fc$^+$	91	/	H-cell	13
250 mM [BMIM][PF_6]/ H_2O/MeCN	Cu_2O-Bi_2O_3	-1.5 V *vs* RHE	95.32	10.68	H-cell	14
30%wt [BMIM][PF_6]/ MeCN	Fe_5Sn_1NC	-2.6 V *vs* Ag/Ag$^+$			H-cell	This work

Table S2. Mass percentages of Fe and Sn determined by ICP-MS, the corresponding metal molar ratios calculated from their mass percentages, and the assigned catalyst names (NC: the support)

Feed molar ratio (Fe:Sn:NC)	Fe (wt %)	Sn (wt %)	Real metal molar ratio (Fe:Sn)	Catalyst name
0.5:1.5:100	0.07 (0.68)[a]	0.07 (0.32)[a]	2:1	Fe_2Sn_1NC
1:1:100	0.22 (0.82)[a]	0.10 (0.18)[a]	5:1	Fe_5Sn_1NC
1.5:0.5:100	0.83 (0.98)[a]	0.03 (0.02)[a]	58:1	$Fe_{58}Sn_1NC$

[a] The data in the parentheses represents molar percentage.

Table S3. Optimization of the feed molar ratio of "metal-to-support" in CO_2RR performance

Feed molar ratio (Fe:Sn:NC)	Potential (V *vs* Ag/Ag$^+$)	FE_{CO} (%)	FE_{H2} (%)	Current density (mA cm^{-2})
1:1:50	-2.4	85.5	0.6	80.9
1:1:75	-2.4	95.1	1.2	59.5
1:1:100	-2.4	98.4	1.3	68.6
1:1:200	-2.4	92.2	1.8	72.3
1:1:400	-2.4	85.0	3.5	31.2

Table S4. Optimization of the feed molar ratio of "Fe to Sn" in CO_2RR performance

Feed molar ratio (Fe:Sn:NC)	Potential (V *vs* Ag/Ag^+)	FE_{CO} (%)	FE_{H2} (%)	Current density (mA cm^{-2})
0.5:1.5:100	-2.4	85.4	5.2	41.7
1:1:100	-2.4	98.4	1.3	68.6
1.5:0.5:100	-2.4	84.7	6.1	33.3

Table S5. Optimization of temperature for the preparation of the Fe/Sn/NC catalyst with a feed molar ratio of 1:1:100

Temperature (℃)	Potential (V *vs* Ag/Ag^+)	FE_{CO} (%)	FE_{H2} (%)	Current density (mA cm^{-2})
700	-2.4	89.4	2.1	66.6
800	-2.4	98.4	1.3	68.6
900	-2.4	72.5	7.2	32.1
1000	-2.4	76.8	6.9	27.9

Table S6. Bader Charge of Fe and Sn in the catalyst before and after the adsorption of key intermediates

Bader charge	Fe	Sn
Before the adsorption	+0.84	+0.46
After the adsorption of *COOH on Fe site	+0.95	+0.50
After the adsorption of *OCHO on Sn site	+0.89	+0.57

4. References

(1) Min, Z.; Chang, B.; Shao, C.; Su, X.; Wang, N.; Li, Z.; Wang, H.; Zhao, Y.; Fan, M.; Wang, J. Enhancing CO_2 electroreduction to syngas by active protons of imidazolium ionic liquids: From performance to mechanism. *Appl. Catal. B Environ.* **2023**, *326*, 122185.

(2) Sun, X.; Lu, L.; Zhu, Q.; Wu, C.; Yang, D.; Chen, C.; Han, B. MoP Nanoparticles Supported on Indium-Doped Porous Carbon: Outstanding Catalysts for Highly Efficient CO_2 Electroreduction. *Angew. Chem. Int. Ed.* **2018**, *57*, 2427-2431.

(3) Gao, S.; Sun, Z.; Liu, W.; Jiao, X.; Zu, X.; Hu, Q.; Sun, Y.; Yao, T.; Zhang, W.; Wei, S.; Xie, Y. Atomic layer confined vacancies for atomic-level insights into carbon dioxide electroreduction. *Nat. Commun.* **2017**, *8*, 14503.

(4) Wei, X.; Li, Z.; Jang, H.; Kim, M.; Liu, S.; Cho, J.; Liu, X.; Qin, Q. Switching Product Selectivity in CO_2 Electroreduction via Cu−S Bond Length Variation. *Angew. Chem. Int. Ed.* **2024**, *63*, No. e202409206.

(5) Valipour, A.; Hamnabard, N.; Meshkati, S.; Pakan, M.; Ahn, Y. Effectiveness of phase- and morphology-controlled MnO_2 nanomaterials derived from flower-like δ-MnO_2 as alternative cathode catalyst in microbial fuel cells. *Dalton Trans.* **2019**, *16*, 5429-5443.

(6) Kresse, G. Ab initio molecular dynamics for liquid metals. *J. Non-Cryst. Solids.* **1995**, *192-193*, 222-229.

(7) Kresse, G.; Hafner, J. Ab initio molecular-dynamics simulation of the liquid-metal-amorphous-semiconductor transition in germanium. *Phys. Rev. B.* **1994**, *49*, 14251-14269.

(8) Monkhorst, H.; Pack, J. Special points for Brillouin-zone integrations. *Phys. Rev. B.* **1976**, *13*, 5188-5192.

(9) Grimme, S., Antony, J., Ehrlich, S., Krieg, H. A consistent and accurate ab initio parametrization of density functional dispersion correction (DFT-D) for the 94 elements H-Pu. *J. Chem. Phys.* **2010**, *132*, 154104.

(10) Wang, V.; Xu, N.; Liu, J.; Tang, G.; Geng, W. VASPKIT: A user-friendly interface facilitating high-throughput computing and analysis using VASP code. *Comput. Phys. Commun.* **2021**, *267*, 108033.

(11) Lv, H.; Dai, C.; Xu, R.; Wang, N.; Chen, B.; Yu, G. In-depth understanding on the mechanism of ionic liquid-assisted enhancement of electrochemical CO_2 reduction to formic acid. *Int. J. Hydrogen Energy*. **2025**, *104*, 103-113.

(12) Mantovi, P.; Cruz, J.; Angelis, L.; Souza, M.; Rossi, L.; Torresi, S.; Lima, F.; Torresi, R. Tuning CO_2RR Pathways through Water Control in Ionic Liquids. *ACS Catal.* **2025**, *15*, 18261-18269.

(13) Vichou, E.; Daura, A.; Draznieks, C.; Li, Y.; Mingot, M.; Fontecave, M.; Sánchez, C. Electrocatalytic Conversion of CO_2 to Formate at Low Overpotential by Electrolyte Engineering in Model Molecular Catalysis. *ChemSusChem.* **2022**, *15*, No. e202201566.

(14) Lv, H.; Guo, F.; Li, J.; Ding, C.; Bi, Z.; Zhang, X.; Zhang, Y.; Chen, B.; Guo, X.; Yu, G. Reverse Reaction Pathways for Efficient CO2-to-Formic Acid Conversion at Cu2O-Bi2O3 Interfaces in Ionic Liquids. *ACS Catal.* **2026**, *16*, 7633-7645.
